# Fatty acid desaturase 1 (FADS1) is a cancer marker for patient survival and a potential novel target for precision cancer treatment

**DOI:** 10.3389/fonc.2022.942798

**Published:** 2022-08-15

**Authors:** Gioia Heravi, Hyejeong Jang, Xiaokun Wang, Ze Long, Zheyun Peng, Seongho Kim, Wanqing Liu

**Affiliations:** ^1^ Department of Pharmaceutical Sciences, Eugene Applebaum College of Pharmacy and Health Sciences, Wayne State University, Detroit, MI, United States; ^2^ Biostatistics and Bioinformatics Core, Department of Oncology, Karmanos Cancer Institute, Wayne State University, Detroit, MI, United States; ^3^ Department of Pharmacology, Wayne State University School of Medicine, Detroit, MI, United States; ^4^ Department of Oncology, Wayne State University School of Medicine, Detroit, MI, United States

**Keywords:** FADS1, PUFA, predictor, survival, TCGA

## Abstract

Fatty Acid Desaturase-1 (FADS1) or delta 5 desaturase (D5D) is a rate-limiting enzyme involved in the biosynthesis of long-chain polyunsaturated fatty acids (LC-PUFAs), i.e., arachidonic acid (ARA) and eicosapentaenoic (EPA). These LC-PUFAs and their metabolites play essential and broad roles in cancer cell proliferation, metastasis, and tumor microenvironment. However, the role of FADS1 in cancers remains incompletely understood. Utilizing The Cancer Genome Atlas (TCGA) database, we explored the role of FADS1 across different cancer types using multiple bioinformatics and statistical tools. Moreover, we studied the impact of a FADS1 inhibitor (D5D-IN-326) on proliferation of multiple cancer cell lines. We identified that FADS1 gene is a predictor for cancer survival in multiple cancer types. Compared to normal tissue, the mRNA expression of FADS1 is significantly increased in primary tumors while even higher in metastatic and recurrent tumors. Mechanistically, pathway analysis demonstrated that FADS1 is associated with cholesterol biosynthesis and cell cycle control genes. Interestingly, FADS1 expression is higher when TP53 is mutated. Tumors with increased FADS1 expression also demonstrated an increased signatures of fibroblasts and macrophages infiltration among most cancer types. Our *in vitro* assays showed that D5D-IN-326 significantly inhibited cell proliferation of kidney, colon, breast, and lung cancer cell lines in a dose-dependent manner. Lastly, single nucleotide polymorphisms (SNPs) which are well-established expression quantitative trait loci (eQTLs) for FADS1 in normal human tissues are also significantly correlated with FADS1 expression in tumors of multiple tissue types, potentially serving as a marker to stratify cancer patients with high/low FADS1 expression in their tumor tissue. Our study suggests that FADS1 plays multiple roles in cancer biology and is potentially a novel target for precision cancer treatment.

## Introduction

Metabolic reprogramming is recognized as one of the hallmarks of cancer metamorphosis (1). The disproportionate growth of tumors results in a limited nutrients availability which makes cancer cells often rearrange their metabolism. Among the nutrients, lipids are a complex group of hydrophobic biomolecules that are crucial for energy metabolism and storage. They also play a significant role in signal transduction (2). Understanding the genetic changes responsible for altered lipid metabolism in cancer will help diagnosis, determination of prognosis, screening and risk assessment, and development of novel targeted treatment (3).

Fatty acids (FAs) are the main building blocks of various lipid species. Cancer cells require a constant supply of FAs for cell proliferation and survival (4). Polyunsaturated fatty acids (PUFAs) including linoleic acid (LA, omega-6) and α-linolenic acid (ALA, omega-3) are essential FAs that must be obtained from the diet. They are precursors of numerous important long-chain PUFAs (LC-PUFAs) e.g. arachidonic acid (ARA), eicosapentaenoic acid (EPA), and docosahexaenoic acid (DHA) which are substantial components of cellular membranes and also serve as bioactive molecules in cell signaling, inflammation, and death (5). Moreover, they are incorporated into phospholipids and triglyceride as well as metabolized to various eicosanoids, endocannabinoids, and pro-resolving lipid mediators (6). PUFA metabolism alterations are found to be an important contributor to tumorigenesis and cancer progression (7). Studies have shown that ARA and its downstream eicosanoids metabolites such as prostaglandins play a role in colorectal cancer progression (8, 9). Prostaglandin E2 (PGE2) also induces cell migration and invasion in breast and lung cancers (10, 11). On the other hand, PGE3, the product of EPA, inhibits tumor angiogenesis (12).

Fatty Acid Desaturase-1 (FADS1) is a rate-limiting enzyme in the biosynthesis of LC-PUFAs, i.e. ARA and EPA (13). FADS1 is identified as an independent cancer prognostic factor in a few studies. Jiao et al. reported that increased FADS1 is correlated with higher tumor grade and worse survival in bladder cancer (BLCA) (14). FADS1 was also found to be upregulated in laryngeal squamous cell carcinoma and was associated with poor prognosis (15). However, in non-small cell lung cancer (NSCLC), FADS1 was downregulated in tumor tissue and patients with lower FADS1 had shorter survival (16).

Genetically, *FADS1* gene is located on human chromosome 11q12-q13.1 (17). Single nucleotide polymorphisms (SNPs) at the locus have been associated with FADS1 gene expression as well-established expression quantitative trait loci (eQTL) among the majority of human tissue and organs (18). These SNPs were also among the most significant genetic determinants for PUFA levels in both human blood and tissues (19–23). Genome-wide association studies (GWAS) have demonstrated that these SNPs are significantly associated with numerous diseases and traits including inflammatory disorder (24), cardiovascular disease (25–27), attention-deficit hyperactivity disorder (ADHD) (28), blood low-density lipoprotein cholesterol and triglyceride levels (29, 30), etc. Interestingly, among these GWAS, rs174548 is strongly correlated with lung cancer risk (31), while rs174537 shows to be correlated with concentration of ARA in prostate tumor tissue and suggests a potential role in prostate cancer risk (32). Another GWAS study in East Asians with colorectal cancer (CRC), identified rs174550 to be associated with CRC risk (33). These studies strongly suggested that FADS1 plays an important role in cancer susceptibility and cancer biology. However, the role of FADS1 in different cancers has not been systematically explored.

In this study, by using the TCGA database, we performed a comprehensive analysis to investigate the role of FADS1 as a key player in 32 types of cancers. Our study demonstrated that FADS1 is a marker for cancer survival, which is potentially driven by a unique molecular mechanism. Our *in vitro* assays demonstrated that pharmacologically inhibiting FADS1 function indeed reduced cancer cell proliferation, suggesting that FADS1 could be a new target for cancer treatment. Our genetic analysis further suggested that FADS1 genotypes can be a potential tool for patient stratification.

## Result

### FADS1 is a marker for patient survival among multiple cancer types

We aim to examine whether *FADS1* expression is associated with cancer patient survival. By leveraging the TCGA data, we performed survival analyses for 29 cancer types after excluding mesothelioma (MESO), pheochromocytoma and paraganglioma (PCPG), and uterine carcinosarcoma (UCS) that did not have the information about both disease-free survival (DFS) and overall survival (OS). The patients’ baseline characteristics downloaded from TCGA database are listed in supplementary materials (**Table S1**). The mean level of the *FADS1* mRNA expression is 0.034 (SD 1.173). The median age at diagnosis is 60 years (range, 10-90 years). The race is defined by two categories: Caucasian and non-Caucasian. 72% of patients were Caucasian. The detailed information by cancer type is included in supplementary materials (**Table S2**).

**Figure 1:**
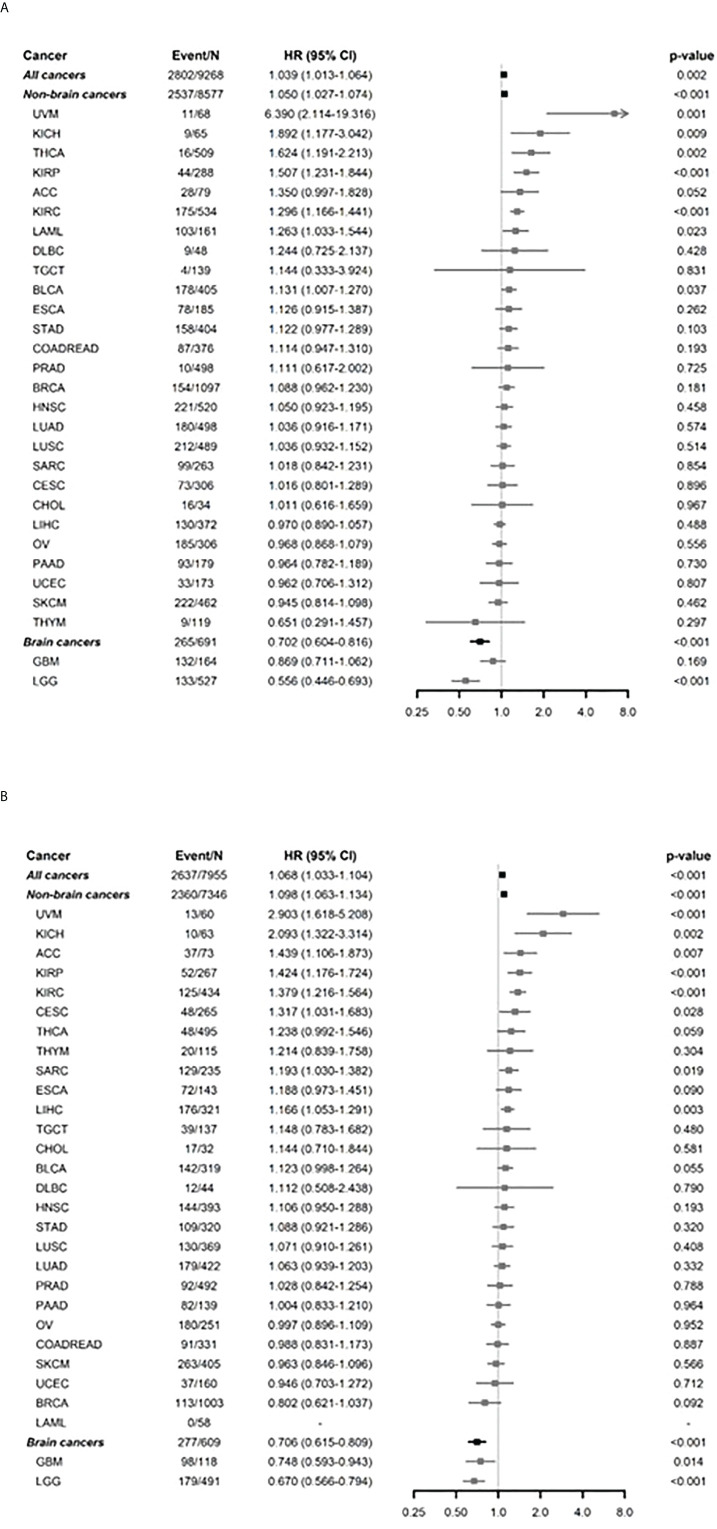
Subgroup analysis by each of cancer types for FADS1 on survival. **(A)** overall survival (OS) after adjusting age and race. **(B)** Disease free survival (DFS) after adjusting age and race. ‘Non-brain cancers’ and ‘Brain cancers’ show the overall summaries for non-brain cancer types and brain cancer types (such as GBM and LGG), respectively. The hazard ratios (HRs) and 95% confidence intervals (CIs) were estimated using multivariable Cox regression analyses between OS and continuous expression levels of FADS1 after adjusting age and race. The HRs and 95% CIs of ‘All cancers’, ‘Non-brain cancers’, and ‘Brain cancers’ were estimated using mixed-effects Cox regression models to account for the effect of different cancer types. The x-axis represents HR.

The associations between *FADS1* expression and survival outcomes of patients with different tumors were further investigated by univariable and multivariable Cox regression and subgroup analyses. The age and race-adjusted hazards ratios (HR) associated with FADS1 mRNA expression in all samples were 1.039 [95% confidence interval (95% CI), 1.013-1.064] and 1.068 [95% CI, 1.033-1.104], for OS and DFS, respectively (**Figures 1A**, **B**; **Table S3** and **S4**). This comprehensive analysis indicated that among all cancer patients, those with a higher FADS1 mRNA expression in their tumor have a significantly worse OS and DFS (P= 0.002 and P<0.001, respectively). This association was particularly significant for OS in Uveal Melanoma (UVM, P=0.001), Kidney Chromophobe (KICH, P=0.009), Thyroid carcinoma (THCA, P=0.002), Kidney renal papillary cell carcinoma (KIRP, P<0.001), Kidney renal clear cell carcinoma (KIRC, P<0.001), Acute Myeloid Leukemia (LAML, P=0.023), Bladder Urothelial Carcinoma (BLCA, P=0.037) and for DFS in UVM (P<0.001), KICH (P=0.002), Adrenocortical carcinoma (ACC, P=0.007), KIRP (P<0.001), KIRC (P< 0.001), Cervical squamous cell carcinoma and endocervical adenocarcinoma (CESC, P=0.028), Sarcoma (SARC, P=0.019), as well as Liver hepatocellular carcinoma (LIHC, P=0.003).

Interestingly, the higher FADS1 expression was significantly associated with better OS (HR=0.702; 95% CI 0.604-0.816; P<0.001) and DFS (HR=0.706; 95% CI 0.615-0.809; P<0.001) among all brain cancers which include Lower grade glioma (LGG) and Glioblastoma multiforme (GBM). Among all non-brain cancers, the HR was 1.050 (95% CI 1.027-1.074; P<0.001) for OS and 1.098 (95% CI 1.063-1.134; P<0.001) for DFS after adjusting age and race.

**Figure 2:**
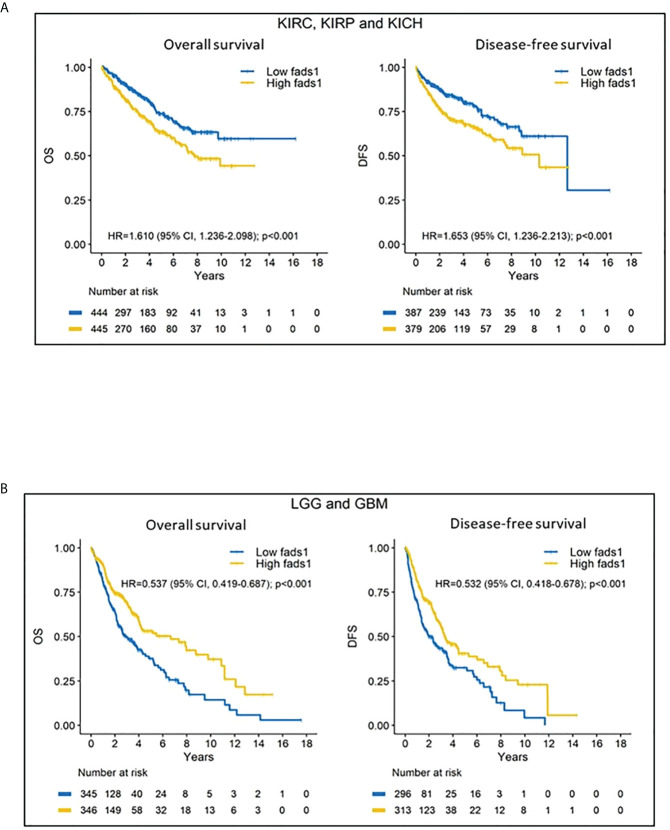
**(A)** Kaplan-Meier (KM) curves of kidney cancers. Kidney cancer patients’ overall survival and disease-free survival according to their FADS1 expression status. **(B)** Kaplan-Meier (KM) curves of brain cancers. Brain cancer patients’ overall survival and disease-free survival according to their FADS1 expression status.

Univariable Kaplan-Meier analyses showed that high FADS1 expression is associated with worse OS (HR=1.610; 95% CI 1.236-2.098; p<0.001) and DFS (HR=1.653; 95% CI 1.236-2.213; p<0.001) for renal cell carcinoma patients (KIRC, KIRP and KICH) (**Figure 2A**), whereas better OS (HR=0.537; 95% CI 0.419-0.687; p<0.001) and DFS (HR=0.532; 95% CI 0.418-0.678; p<0.001) for brain tumor patients (LGG and GBM) (**Figure 2B**). Taken together, our analyses suggested that increased FADS1 expression is associated with worse survival among patients with non-brain cancers, particularly renal cancers, but is associated with better survival among those with brain cancers. The Kaplan-Meier plot for other individual cancer types are included in supplementary materials (**Figures S1**, **S2**).

### FADS1 expression is associated with disease progression

**Figure 3:**
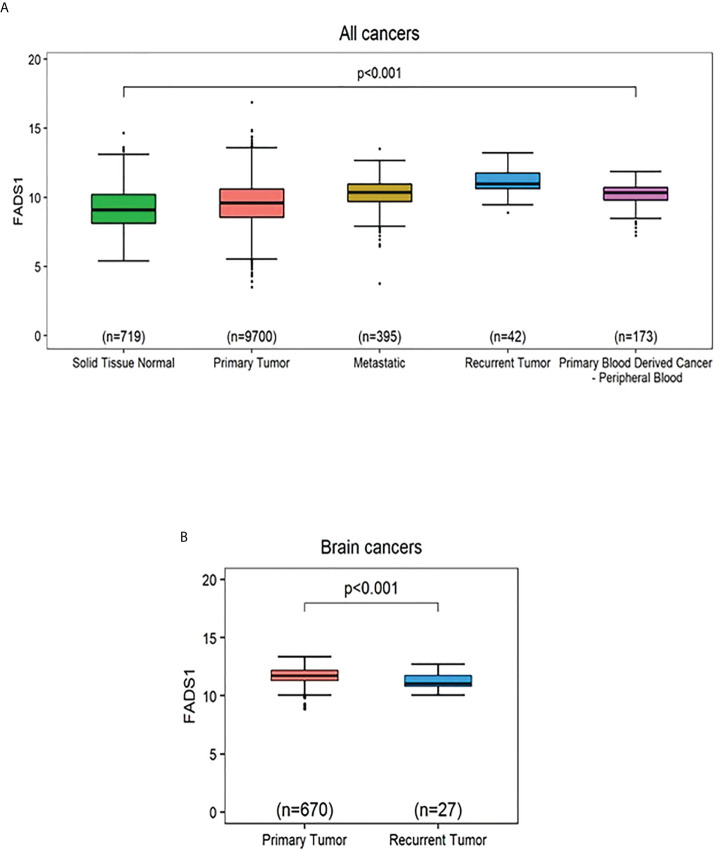
The mRNA expression levels of FADS1 according to sample type normalized by z-score. **(A)** In all cancer types. The median (interval of inter-quartile range [IQR]) is 9.09 (8.13,10.21), 9.58 (8.56,10.60), 10.36 (9.71,10.94), 10.98 (10.64,11.76), and 10.34 (9.81,10.71) for ‘Solid Tissue Normal’, ‘Primary Tumor’, ‘Metastatic’, Recurrent Tumor’, and ‘Primary Blood Derived Cancer - Peripheral Blood’, respectively. The p-value was obtained by Kruskal-Wallis test. All *post-hoc* p values for pairwise comparisons were less than 0.001 except for the pairwise comparison between ‘Metastatic’ and ‘Primary Blood Derived Cancer - Peripheral Blood’ (Wilcoxon *post-hoc* p=0.184). **(B)** In brain cancer types. The p-value was obtained from a Wilcoxon-rank sum test. The median (IQR) is 11.75 (11.31,12.20) and 11.07 (10.82,11.75) for ‘Primary Tumor’ and ‘Recurrent Tumor’, respectively.

We set out to explore the potential mechanism underlying the association between FADS1 expression and patient survival. It is reckoned that metastasis and tumor recurrence are the primary causes of cancer morbidity and mortality (34). We found that FADS1 expression was significantly increased in tumors and especially in metastatic or recurrent tumors than in normal tissues among all cancer samples (**Figure 3A**). This indicates that increased FADS1 expression is associated with tumor recurrence and metastasis, which may be underlying its association with patient survival. Notably, when the samples are analyzed for just brain cancers we found that higher expression of FADS1 is observed in primary tumor compared to recurrent tumors (**Figure 3B**). FADS1 expression levels in each sample type and cancer type, including non-brain cancers, are shown in supplementary materials (**Figure S3**, **S4**). Particularly, increased FADS1 expression is positively associated with tumor formation (primary tumor) and cancer progression (recurrent tumor) among non-brain cancers and negatively associated with cancer progression (recurrent tumor) in brain cancers.

### Patterns of genes co-expressed with FADS1 among different cancer types

**Figure 4:**
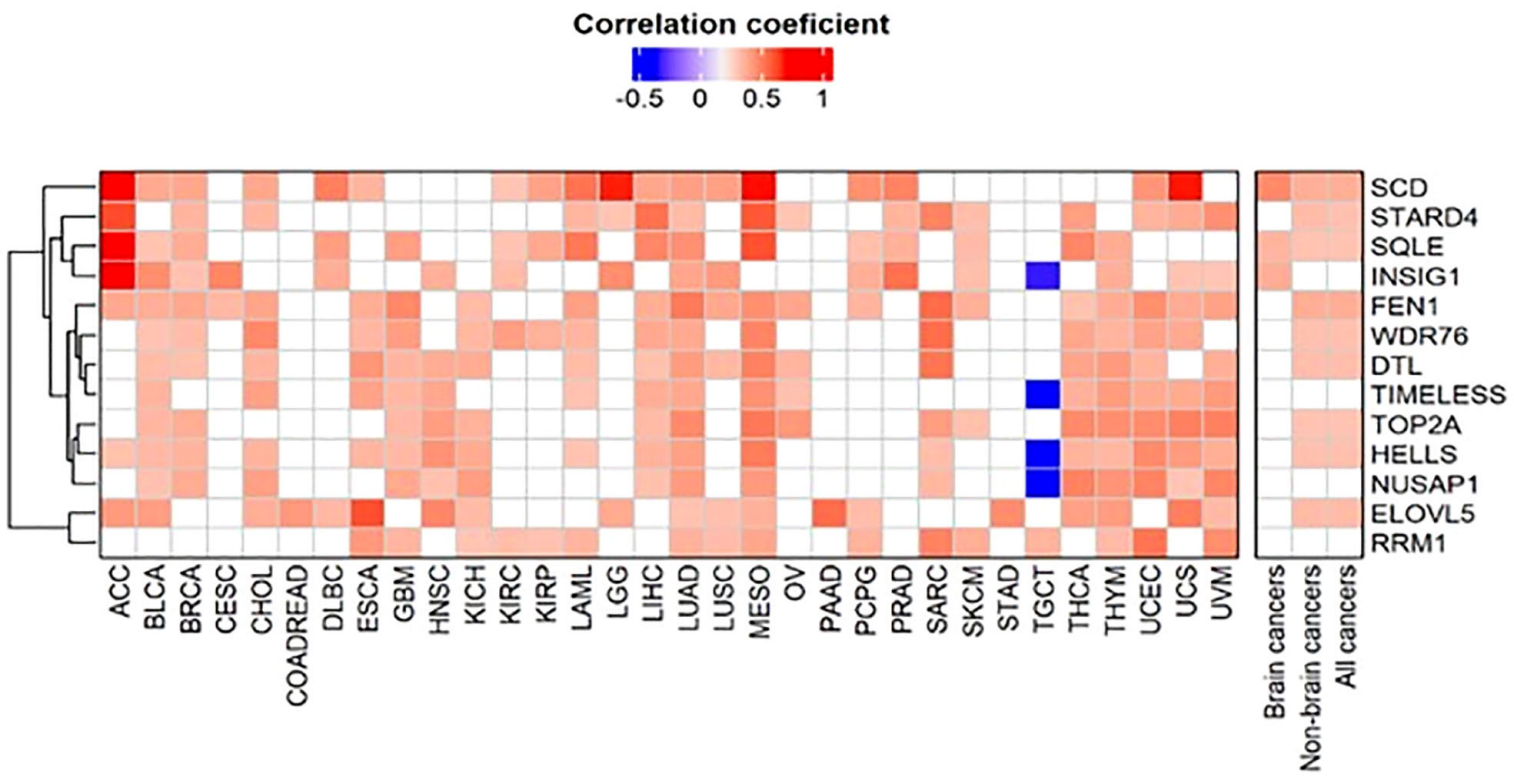
Clustered heatmap of genes correlated with FADS1. The correlations between the RNA expression levels of FADS1 and those of other genes were calculated using Spearman’s correlation. Of genes that have a Spearman’s correlation coefficient ≥0.3 or ≤ -0.3and a p value<0.05, those that were present at least 50% of all cancer types (i.e., 16 cancer types) were selected for the heatmap analysis. For all cancers, non-brain cancers, and brain cancers median was used to summarize correlation coefficient and Fisher’s combined probability test was used for p values. The heatmap is depicted using the Spearman’s correlation coefficients. The empty cell in the heatmap represents that a gene was not correlated with FADS1 in the corresponding cancer type.

To investigate the detailed molecular mechanisms of how FADS1 expression is underlying the associations discovered above, we identified genes that are significantly correlated with FADS1 RNA level in all cancers as a whole and in each cancer type, using Spearman’s correlation. Of genes that have a Spearman’s correlation coefficient ≥0.3 or ≤ -0.3 (p<0.05), those that are present in at least 16 cancer types were selected for the heatmap analysis (**Figure 4**). Genes whose mRNA levels are correlated with that of FADS1 in all cancers and non-brain cancers mainly belong to two categories: lipid metabolism and DNA damage response. Among the former, *SCD* (Stearoyl-CoA Desaturase), *SQLE* (Squalene Epoxidase), *STARD4* (StAR Related Lipid Transfer Domain Containing 4), *INSIG1* (Insulin Induced Gene 1) and *ELOVL5* (Fatty Acid Elongase 5) are key genes involved in fatty acids desaturation and cholesterol homeostasis; while among the latter, *FEN1* (Flap Structure-Specific Endonuclease 1), *DTL* (Denticleless E3 Ubiquitin Protein Ligase Homolog), *TIMELESS* (Timeless Circadian Regulator), *TOP2A* (DNA Topoisomerase II Alpha), *HELLS* (Helicase, Lymphoid Specific) and *RRM1* (Ribonucleotide Reductase Catalytic Subunit M1) are involved in DNA damage response and repair as well as DNA replication. The common genes co-expressed with FADS1 among brain cancers exclusively belong to lipid metabolism (**Figure 4**).

### Pathway enrichment analysis

**Figure 5:**
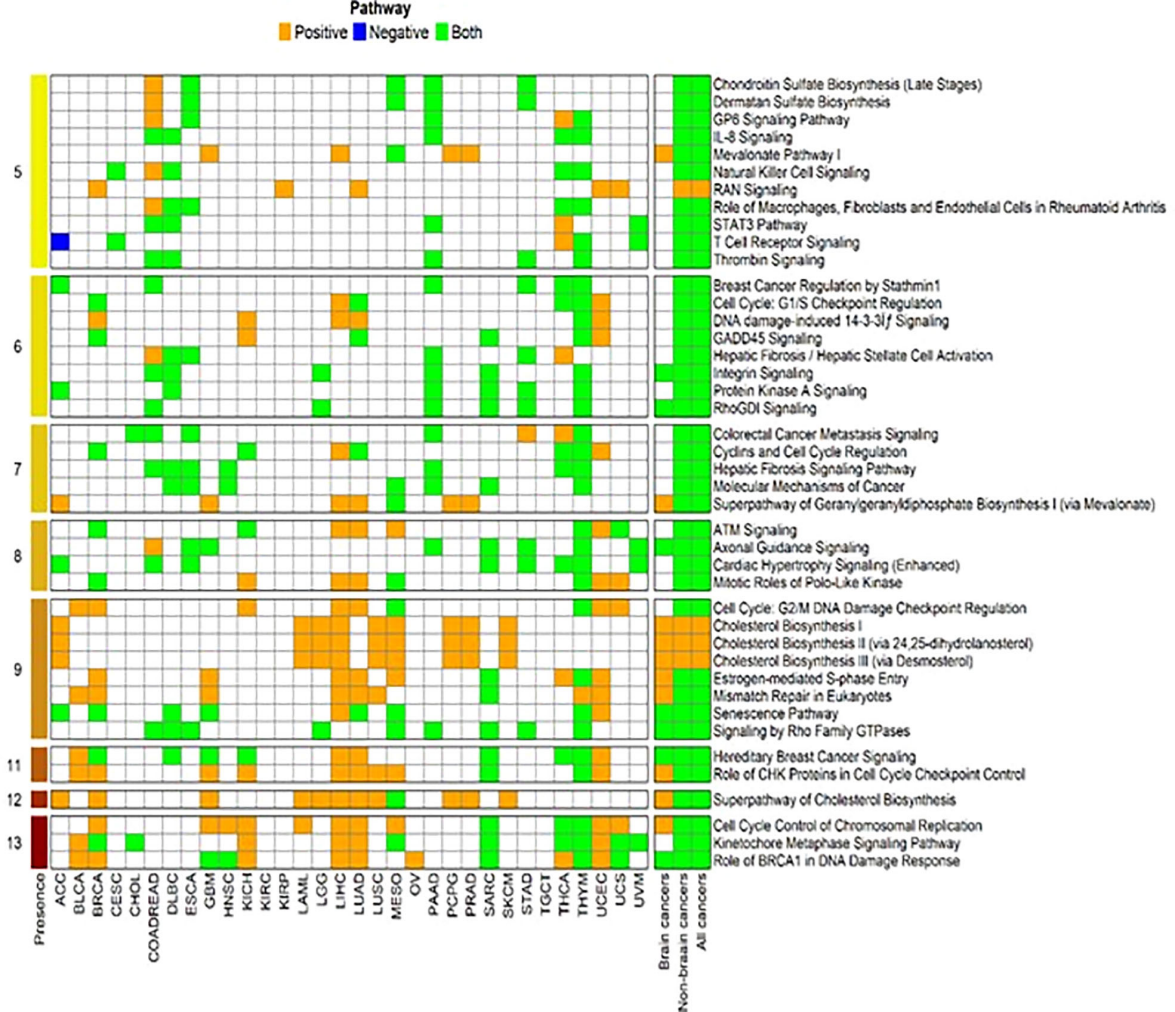
Enriched canonical pathways for genes associated with FADS1. The canonical pathway analysis was performed using Ingenuity Pathway Analysis (IPA, Qiagen, USA) package for each cancer type and the significant pathways were selected at an unadjusted P<0.05. Then we further filtered in the significant canonical pathways that were present among at least 5 individual cancer types. After that, for each cancer type and each selected significant canonical pathway, we identified genes that were a member of the corresponding pathway and were significantly associated with the expression level of FADS1. For each selected pathway, among these identified genes, we indicate as ‘Positive’ if all genes have the positive correlation with FADS1 for each cancer type, ‘Negative’ if all genes have the negative correlation, and ‘Both’ otherwise. The numbers in the leftmost indicate the total number of cancer types where the corresponding significant canonical pathway was present.

The genes correlated with FADS1 with Spearman’s correlation coefficient ≥0.3 or ≤ -0.3 (P <0.05) in each cancer type, brain cancers, non-brain cancers and in all cancers as a whole were used for further pathway enrichment analysis. Canonical pathways that were identified in at least 5 cancer types were further selected for graphical representation (**Figure 5**). Role of BRCA1 in DNA damage response, kinetochore metaphase signaling, cell cycle control of chromosomal replication and superpathway of cholesterol biosynthesis were among the top pathways presented in most of the cancer types. In brain cancers, while the enriched top pathways remained the same as non-brain cancers, the kinetochore metaphase signaling was not observed. Moreover, correlations among non-brain and all cancers are mostly bidirectional but for brain cancers some of the top pathways e.g., Cell cycle control of chromosomal replication and Role of CHK proteins in cell cycle checkpoint control are only positively correlated with FADS1 in brain cancers (**Figure 5**).

### FADS1 expression is associated with key driver mutations

**Figure 6:**
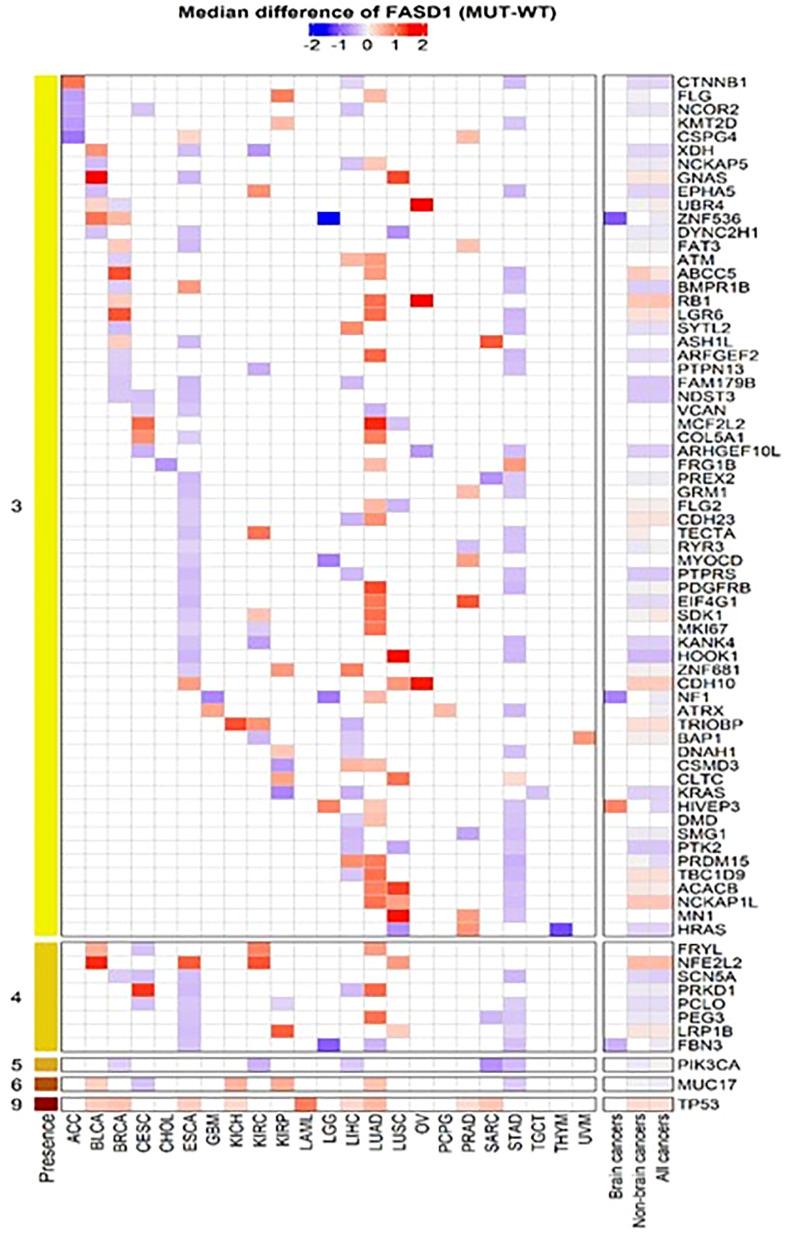
Heatmap of mutated genes associated with FADS1. For each cancer type, we selected genes that had significantly different *FASD1* expression levels between mutation (MUT) and wildtype (WT) in at least three individual cancer types. The Wilcoxon rank-sum test was used to compare the *FADS1* expression levels between MUT and WT for each cancer type.

**Figure 7:**
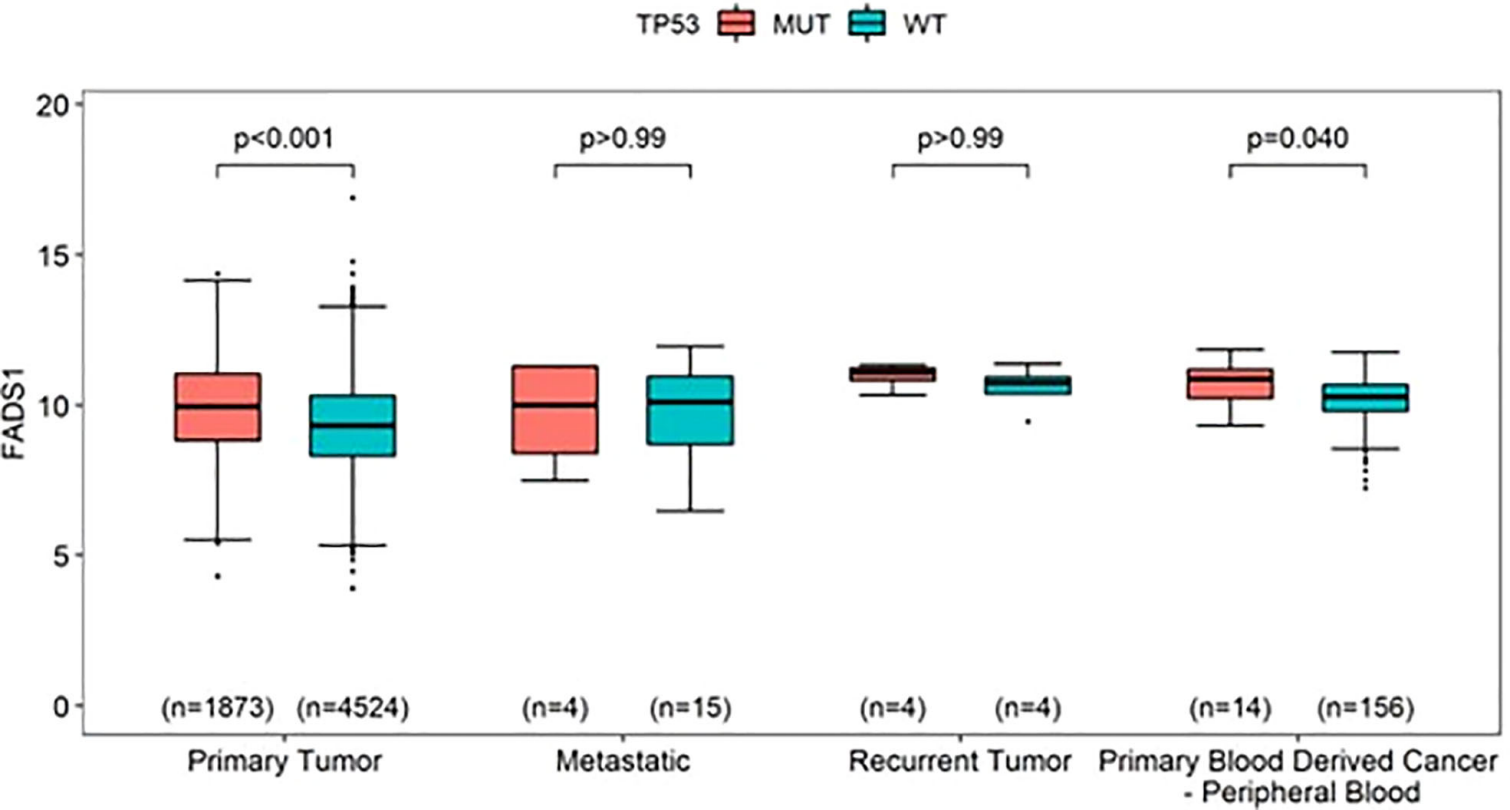
The mRNA expression levels of FADS1 according to sample type and TP53’s mutation status (mutated [MUT] *vs*. wild-type [WT]) in all cancer types. The numbers in parentheses indicate the sample sizes and the p-values were obtained from *post-hoc* Wilcoxon-rank sum tests.

To further explore the relationship between FADS1 gene expression and key cancer driver mutations, genes with mutations in at least 5 samples in each cancer type were selected to assess their associations with the mRNA expression levels of FADS1. The genes that had significantly different RNA expression levels of FADS1 between mutated and wild-type in at least one individual cancer type were considered. (**Figure 6**). The most prevalent mutation across individual cancer types was mutated *TP53* that was significantly correlated with higher RNA expression of FADS1 among 9 individual cancer types, non-brain cancers, and all cancers but not brain cancers. On the other hand, *PIK3CA* mutations were correlated with lower FADS1 RNA expression. These data indicate that there may be a reciprocal interaction between FADS1 function and key tumor driver mutations. Additionally, we continued to investigate the relationship between *FADS1* mRNA expression and *TP53* mutation status in different sample type. Among primary tumors, higher *FADS1* mRNA expression was significantly correlated with *TP53* mutations (P<0.001) (**Figure 7**). This association was also significant in primary blood-derived cancers (P=0.04). In metastatic and recurrent tumors, the relationship was not significant (**Figure 7**). In brain cancers the *TP53* mutation status in primary tumors and recurrent tumor was not significantly associated with *FADS1* mRNA level (**Figure S5**).

### FADS1 expression is associated with tumoral infiltration of immune cells

The tumor microenvironment (TME) plays a crucial role in tumorigenesis and cancer progression (35). One of the major components of TME are immune cells such as macrophages and tumor stromal cells including stromal fibroblasts (36). Tumor associated macrophages (TAMs) contribute to cancer-related inflammation (37). Cancer associated fibroblasts (CAFs), are associated with ECM remodeling, recruiting immune cells, modulating their function and promoting cancer progression (38). The direct metabolic products of FADS1, ARA and EPA are key molecular precursors of various signaling lipids involved in regulating immunity and inflammatory response (39). We thereby examined the hypothesis that FADS1 expression is correlated with tumoral immune cell infiltration.

**Figure 8:**
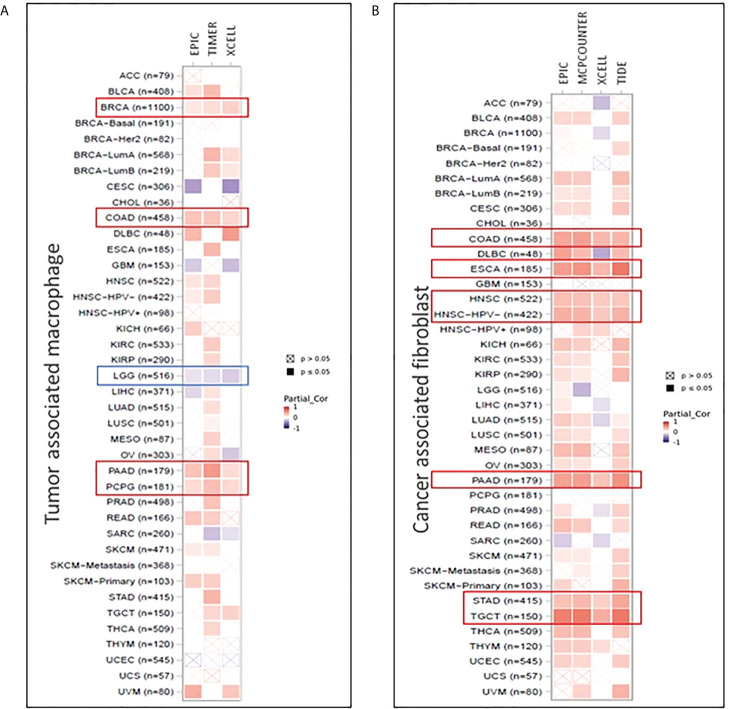
Correlation between FADS1 and immune signatures in TIMER 2.0. **(A)** Tumor associated macrophage infiltration in correlation with FADS1 in TCGA cancer types. **(B)** Cancer associated fibroblast infiltration in correlation with FADS1 in TCGA cancer types.

Herein, we used TIMER2.0 tool (40) to demonstrate the relationship between immune cell filtration and FADS1 expression in TCGA cancer types. Positive correlation between FADS1 expression and increased macrophage infiltration was of note particularly in breast invasive carcinoma (BRCA), colon adenocarcinoma (COAD), pancreatic adenocarcinoma (PAAD), and pheochromocytoma and paraganglioma (PCPG) cancer types with significant correlation demonstrated with all three EPIC, TIMER and XCELL algorithms. On the contrary, negative correlation was observed in LGG and a similar trend was observed in GBM (**Figure 8A**). There was also a significant positive correlation between FADS1 expression and CAF abundance in multiple cancer types. The strongest correlation was observed in COAD, esophageal carcinoma (ESCA), head and neck squamous cell carcinoma (HNSC), PAAD, stomach adenocarcinoma (STAD) and testicular germ cell tumors (TGCT) (**Figure 8B**). This result suggests that increased FADS1 expression is also important for TME, which may further contribute to tumor metastasis and/or recurrence.

### FADS1 SNPs are significantly associated with FADS1 expression levels in tumors

**Figure 9:**
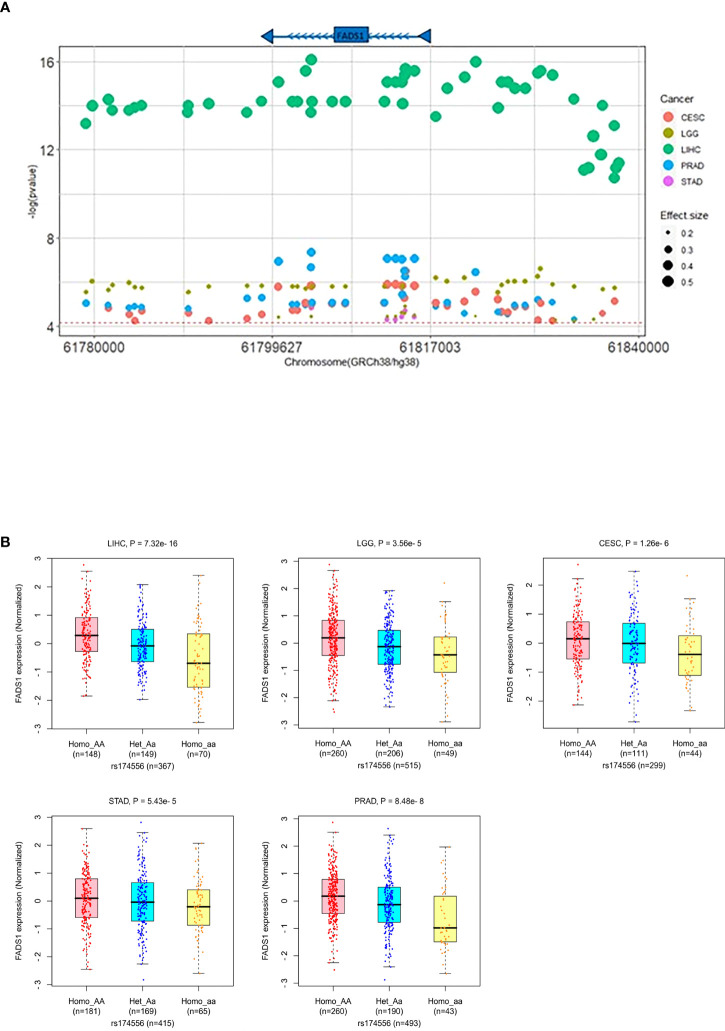
**(A)** FADS1 eQTLs in the FADS1 locus (+/-500kb) in the related cancer type. The horizontal red line represents the threshold for Bonferroni-corrected significance (P<7.05×10-5). The color of dots represents the cancer type and the size represents the effect size (beta). **(B)** Association between a typical FADS1 eQTL rs174556 and FADS1 mRNA expression among different cancer type(generated in PancanQTL). P values were computed based on ANOVA analysis.

Given the association between FADS1 expression and patient survival, it is important to have readily available markers to predict the FADS1 expression in tumor cells. It has been well-established that SNPs across the FADS1-2 locus are strong eQTLs of FADS1 among human normal tissues. We examined whether SNPs around the locus are also eQTLs predictive of the tumoral FADS1 mRNA expression. A total of 709 unique eQTLs (associated with *FADS1* mRNA expression in at least one cancer type with a nominal P<0.05) for FADS1 have been identified in TCGA among all cancer types (**Figure S6**). After bonferroni-correction (P<7.05×10^-5^), 81 SNPs were signficantly associated with *FADS1* mRNA exression mainly among 5 cancer types: CESC, LGG, LIHC, prostate adenocarcinoma (PRAD) and STAD (**Figure 9A**). The highest and lowest effect size were observed in LIHC and LGG, respectively. It is known that there is a strong linkage disequilibrium among these SNPs (19). We plotted the correlation between a representative SNP rs174556 and FADS1 mRNA levels among these tumor types as an example (**Figure 9B**).

### Inhibiting FADS1 reduced cancer cell proliferation *in vitro*

**Figure 10:**
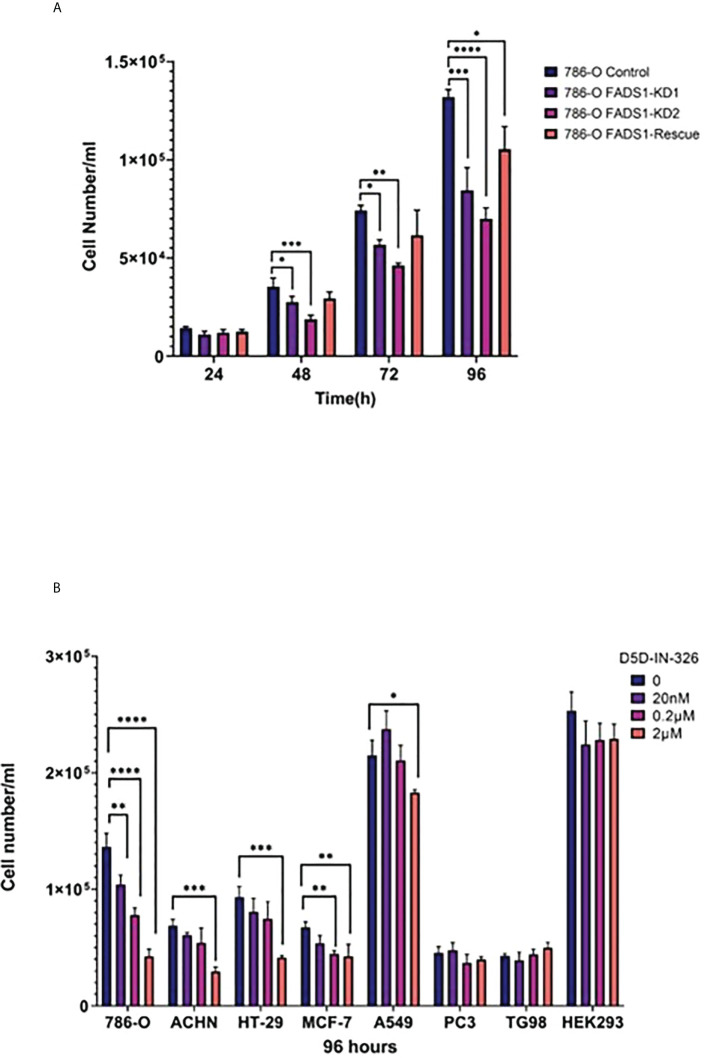
*In vitro* Study on FADS1. **(A)** Cell proliferation after FADS1 knockdown (KD) in 786-O cell line. **(B)** Cell proliferation in different cell lines. FADS1 inhibitor treatment in cancer cell lines at different doses after 96-hour treatment with FADS1 inhibitor. All experiments were performed in triplicates. *P<0.05, **P<0.01, ***P < 0.001, ****P<0.0001.

To further validate whether FADS1 is causally involved in cancer cell proliferation, we chose a renal cell carcinoma cell line 786-O since *FADS1* mRNA level shows the most significant correlation with patient survival in kidney cancers (**Figures 1A**, **B**). We knocked down FADS1 expression in 786-O cells using shRNA. We also generated a rescue cell line by reintroducing *FADS1* full sequence in KD cells to restore the phenotype (**Figure S7**). Later, we conducted a proliferation assay on the stable cell lines. The proliferation of 786-O cells with reduced *FADS1* expression was significantly downregulated as compared to the control and rescue cell lines. (**Figure 10A**).

To examine the anti-cancer potential of pharmacological inhibition of FADS1, we used a FADS1 inhibitor (D5D-IN-326) to examine the cell proliferation in cell lines of multiple cancer types, including renal (786-O, ACHN), colon (HT-29), lung (A549), breast (MCF-7), prostate (PC3) and glioblastoma (T98G) cell lines (**Figure 10B**). We also used HEK293, a non-cancerous embryonic kidney cell line. The inhibitor was able to hinder cell proliferation in all cancer cell lines significantly except PC3 and T98G. Among different cancer cell lines, 786-O cell line was the most sensitive to the inhibitor. Interestingly, the inhibitor was not able to significantly reduce the cell proliferation of HEK293. We also evaluated the effect of D5D-IN-326 (2µM) in 786-O stable cell lines. The anti-proliferative effect of D5D-IN-326 was observed in both control and the rescued cells but not in the FADS1-KD cells (**Figure S8**).

Taken together, our data suggested that FADS1 is causally involved in cancer cell proliferation, thus can be a pharmacological target for anti-cancer treatment.

### Transcriptomic alterations associated with FADS1 inhibition

To confirm the causal role of FADS1 in altering transcriptomic signatures we have observed from the TCGA data, we carried out RNA sequencing analysis for the FADS1 knockdown 786-O cell line. There were 958 genes showing significant change in their expression level in FADS1 knockdown cells as compared to the control cells (FDR<0.05). The top 20 canonical pathways enriched among these genes are shown in **Figure S9**. The complete list of pathways is provided in **supplementary data file S1**. Overall, the pathways enriched among these genes were highly similar to those enriched among the genes co-expressed with FADS1 in the TCGA data (**Figure S9**), i.e. cell cycle regulation cell proliferation and DNA damage repair pathways were the top ones among both datasets.

## Discussion

Increasing evidence has suggested that FADS1 and its controlled LC-PUFA metabolism play important role in cancer risk, biology and progression. Our study for the first time demonstrated that FADS1 is a marker correlated with prognostic outcomes of cancer patients in multiple cancer types. Our detailed data analyses indicated that tumoral FADS1 mRNA level is significantly associated with cancer metastasis and recurrence, which is possibly driven by its role in cell cycle control *via* interacting with multiple pathways e.g. P53 and PI3KCA. Moreover, FADS1 may also play an important role in modulating the tumor microenvironment. Our *in vitro* assay further validated the causal role of FADS1 in regulating cancer cell proliferation and suggested that pharmacological inhibition of FADS1 can be a novel strategy for anti-cancer treatment. Lastly, we further demonstrated that tumoral expression of FADS1 is significantly associated with genotypes of the SNPs in the FADS1 gene locus, which provides a potential strategy for pre-screening of patients suitable for FADS1-targeted treatment.

Our analyses demonstrated that *FADS1* mRNA level in the tumor is significantly associated with cancer patient survival. This association varies among different cancer types, with major kidney cancer types showing the most significant associations. Interestingly, all brain tumor types demonstrated a negative correlation between *FADS1* expression and patient survival. The reason for this difference is unknown. The co-expression gene profiles as well as the TME-related tumoral cell infiltration data also demonstrated an opposite direction in their association with *FADS1* expression between kidney cancers and brain tumors. Also, the *in vitro* assay demonstrated that RCC cells and brain cancer cells are sensitive and resistant to FADS1 inhibitor, respectively. These data together indicated that FADS1 may function in different mechanisms between brain and non-brain cancers when it is involved in cancer biology.

We found that *FADS1* mRNA level was significantly increased in tumors, especially in metastatic or recurrent ones, than that in normal tissues among all cancers (**Figure 3A**). This might indicate that increased *FADS1* expression is associated with tumor aggressiveness, which may explain at least in part, its association with patient survival. Meanwhile, our analysis showed that *TP53* mutation status. Is associated with higher *FADS1* expression. Garritano et al. (41) conducted a review of literature to identify the genes differentially modulated upon the expression of mutant P53. Their analysis demonstrated that *FADS1* expression is upregulated when P53 is mutated. The tumor suppressor P53 controls cell cycle, regulates DNA repair, and promotes apoptosis (42). Mutations in this gene are observed in more than half of human cancers (43), with the majority of these mutations being loss-of-function. Given the transcriptional regulation function of P53, it is possible that FADS1 is under the direct control of P53. In fact, genome-wide chromatin occupancy and gene expression analyses studies have shown that *FADS1* locus contains P53 binding motifs, which may affect *FADS1* expression upon DNA damage induction (44, 45).

The primary role of FADS1 is the production of ARA in omega-6 series and EPA in omega-3 series of LC-PUFAs. Numerous studies including ours have clearly demonstrated that FADS1 expression directly regulates the production of LC-PUFAs (46). Rapidly proliferating cancer cells demand a high concentration of fatty acids for membrane synthesis, as signaling molecules, and as a source of energy (47). To avoid lipotoxicity FA desaturation *via* FADS1 may be an adaptive strategy that cancer cells use for survival. Meanwhile, both ARA and EPA are important precursors of numerous signaling lipid molecules e.g. prostaglandins, leukotrienes, endocannabinoids, resolvins and maresins, etc. (6). A number of studies have demonstrated the key roles of these molecules in modulating cell proliferation, growth, and apoptosis in cancer (48–50). On the other hand, LC-PUFAs are important components of phospholipids in biomembranes, which control the membrane fluidity and affect membrane protein function, e.g. signaling molecules (51, 52). Cell membrane fluidity altered in malignant cells can facilitate the changes in their plasticity and motility. This feature is controlled with two distinct parameters: cholesterol content of the membrane that makes it rigid, and unsaturated phospholipid content that is responsible for the fluidity (53). Indeed, cholesterol biosynthesis pathway is shown to be among the most significantly enriched pathways in the FADS1-coepxressing genes (**Figure 5**). Research is ongoing in our lab to understand the relationship between altered *FADS1* expression changes and membrane properties, as well as the relationship between remodeling of PUFA and cholesterol metabolism that is related to FADS1 function changes.

In addition, the positive association between FADS1 expression and transcriptome profile-predicted TAMs and CAFs infiltrated into tumors may be related to the important role of FADS1 products in immunity and inflammation. ARA is well-known as the precursor for pro-inflammatory mediators, while EPA and DHA are known for their anti-inflammatory properties. These LC-PUFAs along with their metabolites may be involve in modulating inflammation signaling in TME. For example, it is known that infiltrated CAFs causes severe fibrosis called desmoplasia in PAAD (54). In our data *FADS1* expression is indeed positively associated with both increased TAM and CAF signals in PAAD. On the other hand, we found that higher *FADS1* expression is associated with lower TAMs in LGG and GBM, which may reflect the different kinetics of LC-PUFAs between brain and non-brain tissues. Healthy brain tissue is enriched in ARA and DHA. However, in brain tumors DHA level is reduced (55, 56). Increased FADS1 activity in the brain may be responsible for higher level of DHA that can decrease the production of inflammatory cytokines (57). As a result, vascular permeability is reduced and interferes with macrophage infiltration. Nevertheless, this analysis is based on deconvoluted transcriptome data thus should be further validated with histological analyses of human tumor tissues.

Our *in vitro* studies demonstrated that reduced *FADS1* expression or activity is indeed causally integral to cancer cell proliferation. Meanwhile, pharmacological inhibiting FADS1 activity also demonstrated a dose-dependent response among non-brain cancer cells, and not in non-cancerous cells, suggesting that FADS1 is a potential novel target for anti-cancer treatment. To this end, patients with increased *FADS1* expression in their tumor (decreased in brain tumor) could be more likely to benefit from FADS1-targeted treatment. We found that known eQTLs of FADS1 gene among five cancer types still remain to be its eQTLs in tumor tissues. These eQTLs are in strong linkage disequilibrium and studies showed that these SNPs can predict *FADS1* expression as well as FADS1 activity that is reflected in circulating PUFA levels in humans (19, 58, 59). Change in the level of PUFAs that are due to these genetic variants, are demonstrated to be associated with cancer risk (31, 33, 60, 61). Thus, it is possible that SNP genotyping may be a pre-screening strategy for identifying most suitable patients for such a therapy. The value of these SNPs in predicting responses to FADS1-targeting treatment remains further explored.

In summary, our study highlighted that FADS1 is a key gene broadly involved in cancer risk, biology, and patient prognosis. The new hypotheses generated from our study warrant further validation analyses both *in vitro* and *in vivo*. Once confirmed, this will open a new avenue for a precision targeted anti-cancer therapy.

## Materials and methods

### Reagents

FADS1 inhibitor (D5D-IN-326) was purchased from Sigma-Aldrich (cat no. SML2865). All cell lines were culture and maintained in Dulbecco’s modified Eagle’s medium (DMEM) supplemented with 10% fetal bovine serum (FBS) except for TG98 that was cultured in essential minimum Eagle’s medium (EMEM) with 10% FBS.

### Survival analysis

TCGA provisional data were obtained through cBioPortal (https://www.cbioportal.org/) (62, 63) for age, race, RNA expression levels of FADS1, and survival outcomes (disease-free survival [DFS] and overall survival [OS]). The List of TCGA cancer abbreviation is included in **Table S5**. There were total of 32 cancer types available and, of these, mesothelioma (MESO), pheochromocytoma and paraganglioma (PCPG), and uterine carcinosarcoma (UCS) did not have the information about both DFS and OS. Thus, those three cancer types were excluded for the survival analysis. In addition, there were five patients who had negative values for DFS and OS, which were replaced with NA for survival analysis (two patients in BLCA and one patient in each of LGG, SKCM, and UCEC). The RNA expression levels normalized by z-score were used for the analysis without any transformation. Race was divided into two levels (Caucasian versus Non-Caucasian) and patients who are not Caucasian or have unknown ethnicity were considered Non-Caucasian. The distributions of DFS and OS were graphically summarized using Kaplan-Meier (KM) plots, and comparisons between groups were carried out using a log-rank test. The univariable and multivariable Cox regression analyses were used to see the associations between prechosen covariates (FADS1, age, and race) and survival outcomes (DFS and OS). In particular, to account for the effect of different cancer types, the overall analyses were carried out using mixed-effects Cox regression models. The proportional hazard assumption was checked and no violation was found. The subgroup analyses of DFS and OS were performed for each cancer type and presented their outcomes using forest plots.

### FADS1 expression and disease progression

For disease progression, data were obtained through XENA((https://xenabrowser.net/) (64)for FADS1 expression, Sample type and Cancer type XENA. We selected TCGA Pan-Cancer (PANCAN) study. Among 12840 TCGA patients’ data, 11047 had FADS1 expression values. For the correlation between FADS1 expression and sample type we used Kruskal-Wallis test to obtain the p-value. We have also included the correlation between FADS1 and sample type in each cancer type except for those cancer type that do not have at least two sample type to be compared (**Supplementary Materials**; **Figures S3**, **S4**).

### Genes correlated with FADS1 among different cancer types and pathway analysis

Spearman correlation coefficients were used to assess the associations between the RNA expression level of FADS1 and those of other genes for each cancer type. Genes with a correlation coefficient of ≥0.3 and ≤-0.3 and a p-value of ≤0.05 were selected for the pathway analysis and grouped them into two groups, positive and negative, according to the signs of their correlation coefficients. Canonical pathways were identified using the selected genes for each cancer type using Ingenuity Pathway Analysis (IPA, QIAGEN) with an unadjusted p-value of 0.05 and grouped into three groups, positive, negative, and both, according to the genes included, where both means that both positive and negative genes were included in the corresponding pathway.

### FADS1 expression and driver mutations

Genes with mutated in at least 5 samples were selected to assess the associations with the RNA expression levels of FADS1 for each cancer type. The associations between FADS1 and selected genes were evaluated using Wilcoxon rank-sum test with an unadjusted p-value of 0.05.

### FADS1 expression and tumoral infiltration of immune cells

The relationship between FADS1 expression and immune infiltration was determined using the TIMER2.0 (http://timer.cistrome.org/) (40). Purity adjustment option was selected. Several immune cells were investigated. Macrophage and fibroblast infiltration were among the most significant correlated immune signature cells with FADS1 expression in many cancer types. So, these two infiltrations were chosen for display. We included data generated using TIMER (65), EPIC (66) and XCELL (67) in our analysis to show that FADS1 expression is significantly correlated with the infiltration of macrophages. These algorithms use gene signature-based approach for immune infiltration estimation. TIMER also has the advantage to take tissue specificity into account, while XCELL makes estimations on higher number of cells (65, 67). On the other hand, EPIC can simultaneously estimate the fraction of cancer and immune cell types from bulk tumor gene expression data (66). To study the association between FADS1 expression and CAF abundance in tumor microenvironment we included microenvironment cell populations-counter (MCP-counter) (68), and Tumor Immune Dysfunction and Exclusion (TIDE) (69) algorithms along with EPIC and XCELL.

### FADS1 polymorphism

We collected eQTL information about SNPs around the FADS1 locus (+/-1Mb) based on the TCGA data using PancanQTL (http://gong_lab.hzau.edu.cn/PancanQTL/) (70). A total of 709 unique eQTL for FADS1 has been identified in TCGA with a nominal P<0.05 (**supplementary data file S2**). To identify the signifcant SNPs we used Bonferroni correction (P<7.05 × 10^-5^). Human genome reference GRCh38/hg38 is used to depict the location of SNPs in FADS1 locus in **Figures 9A**, **S6**.

### Cell proliferation study

786-O stable cells with FADS1 knockdown (KD) were constructed using short hairpin RNA (shRNA). To create stable cell lines, seeded cells were incubated at 37°C with 5% CO2 overnight. When cultures reached 70% confluency, the medium was removed, and the lentivirus suspension either FADS1 shRNA lentivirus or control shRNA lentivirus was diluted in complete medium with polybrene (5 µg/mL) in each well. Subsequently, cells were incubated for 24 hours, and the next day, the medium was removed and replaced with complete medium with 10% FBS with puromycin (2 μg/mL) for an additional 48 hours to select for stably transduced cells. The appropriate selection medium was replaced every 3 to 4 days, and selection was performed for at least 14 days. Puromycin-resistant clones were then transferred into a tissue culture flask for further maintenance and experiments. To construct the rescue cell lines, KD stable cell line was incubated with lentiviral vector encoding full FADS1 open reading frame (ORF) diluted in complete medium as explained above. The selection agent for rescue cell line was Hygromycin (200 μg/mL).

To investigate the impact of FADS1 status on cell proliferation, stable cell lines and other cancer cell lines were seeded in 24-well plate at 10^4^cell/ml in triplicate. For D5D-IN-326, three concentrations of 20nM, 0.2µM, 2µM were used. Every 24 hours, cells were harvested and counted with hemocytometer after 96 hours.

## Data availability statement

The original contributions presented in the study are included in the article/**Supplementary Materials**. Further inquiries can be directed to the corresponding author.

## Author contributions

GH, WL, and SK conceptualized and designed the study. GH, HJ, XW, ZL, and ZP contributed to the investigation and data curation. GH, HJ, SK, and WL performed formal analysis and visualization. GH wrote the manuscript. WL and SK supervised the research. All authors contributed to the article and approved the submitted version.

## Funding

This study is supported in part by the NIH/NIDDK R01DK124612 (WL), NIH/NIDDK R01DK106540 (WL), NIH/NCI P30 CA022453 (HJ and SK) NIH/NIGMS R21GM140352 (SK) and an NCI T32-CA009531 Pre-doctoral Training fellowship to GH.

## Acknowledgments

The authors wish to thank Dr. Jing Gong for assistance with FADS1 SNPs information of TCGA database.

## Conflict of interest

The authors declare that the research was conducted in the absence of any commercial or financial relationships that could be construed as a potential conflict of interest.

## Publisher’s note

All claims expressed in this article are solely those of the authors and do not necessarily represent those of their affiliated organizations, or those of the publisher, the editors and the reviewers. Any product that may be evaluated in this article, or claim that may be made by its manufacturer, is not guaranteed or endorsed by the publisher.
