# Supplementary material for: Fatty acid desaturase 1 (FADS1) is a cancer marker for patient survival and a potential novel target for precision cancer treatment
Source: Front Oncol. 2022 Aug 15;12:942798. doi: 10.3389/fonc.2022.942798 (PMC9423679; doi:10.3389/fonc.2022.942798)
Supplement: Supplementary file 1 [file Presentation_1.pptx]

## Slide 1
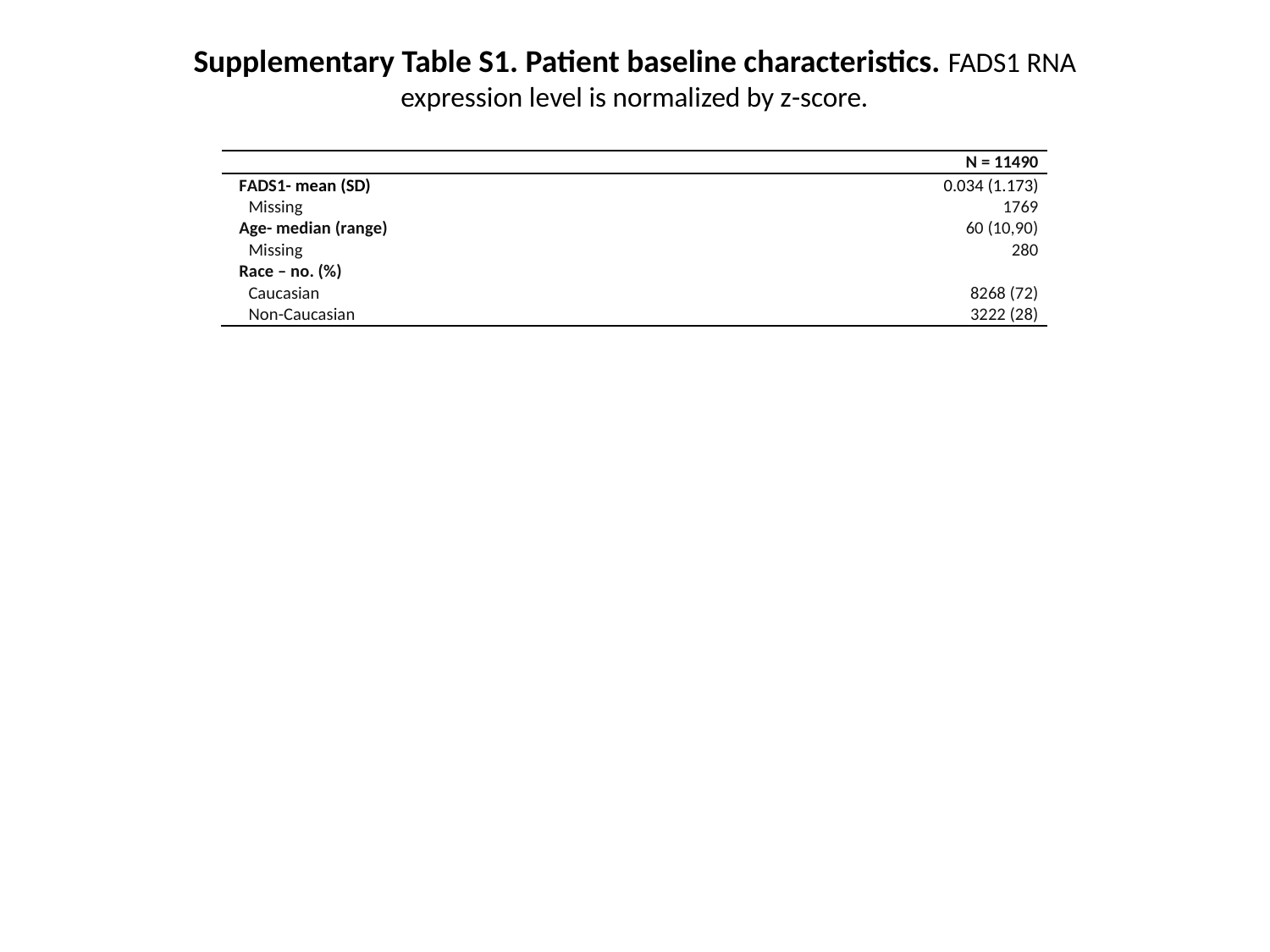

Supplementary Table S1. Patient baseline characteristics. FADS1 RNA expression level is normalized by z-score.

## Slide 2
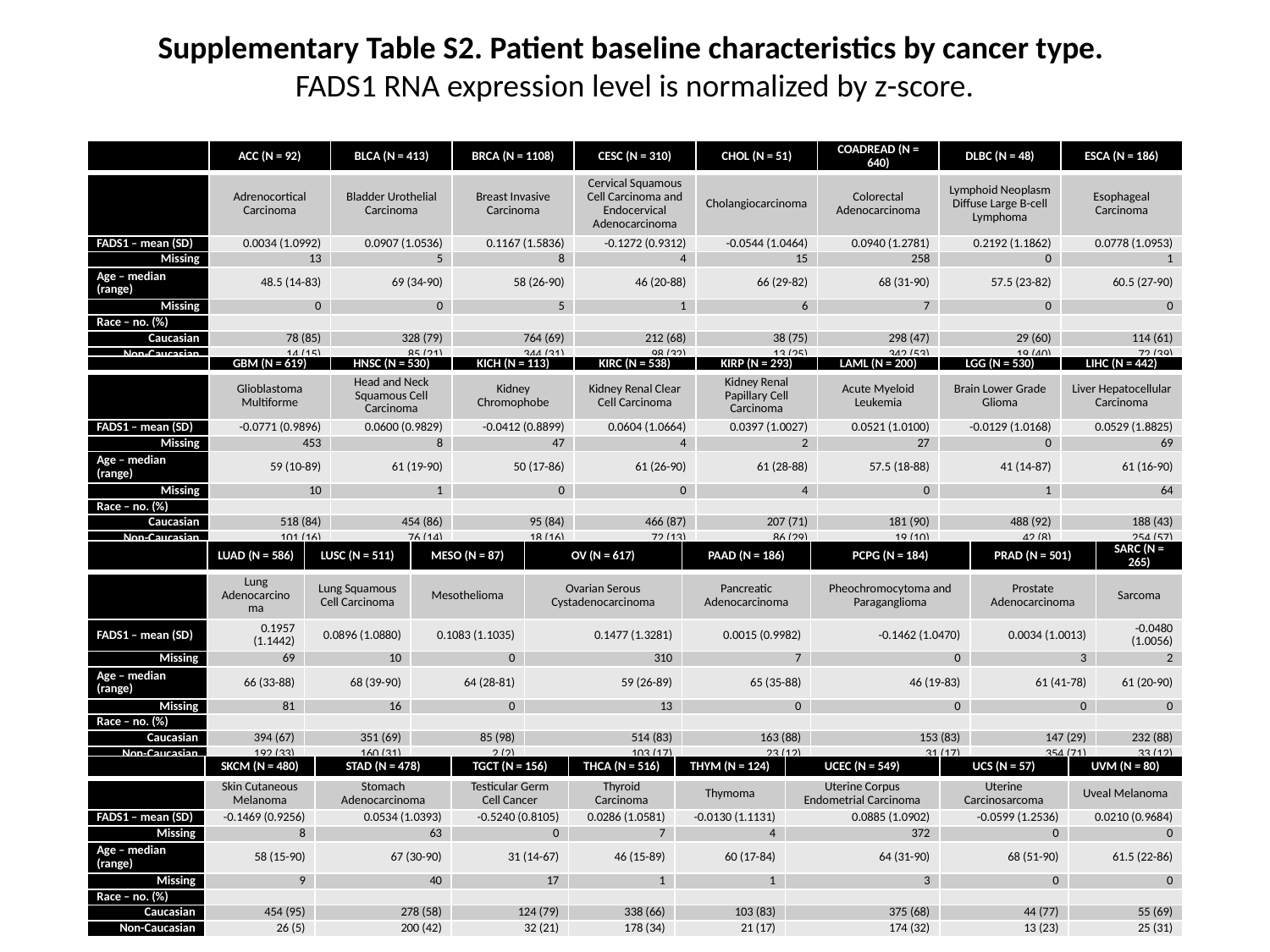

Supplementary Table S2. Patient baseline characteristics by cancer type.
FADS1 RNA expression level is normalized by z-score.
| | ACC (N = 92) | BLCA (N = 413) | BRCA (N = 1108) | CESC (N = 310) | CHOL (N = 51) | COADREAD (N = 640) | DLBC (N = 48) | ESCA (N = 186) |
| --- | --- | --- | --- | --- | --- | --- | --- | --- |
| | Adrenocortical Carcinoma | Bladder Urothelial Carcinoma | Breast Invasive Carcinoma | Cervical Squamous Cell Carcinoma and Endocervical Adenocarcinoma | Cholangiocarcinoma | Colorectal Adenocarcinoma | Lymphoid Neoplasm Diffuse Large B-cell Lymphoma | Esophageal Carcinoma |
| FADS1 – mean (SD) | 0.0034 (1.0992) | 0.0907 (1.0536) | 0.1167 (1.5836) | -0.1272 (0.9312) | -0.0544 (1.0464) | 0.0940 (1.2781) | 0.2192 (1.1862) | 0.0778 (1.0953) |
| Missing | 13 | 5 | 8 | 4 | 15 | 258 | 0 | 1 |
| Age – median (range) | 48.5 (14-83) | 69 (34-90) | 58 (26-90) | 46 (20-88) | 66 (29-82) | 68 (31-90) | 57.5 (23-82) | 60.5 (27-90) |
| Missing | 0 | 0 | 5 | 1 | 6 | 7 | 0 | 0 |
| Race – no. (%) | | | | | | | | |
| Caucasian | 78 (85) | 328 (79) | 764 (69) | 212 (68) | 38 (75) | 298 (47) | 29 (60) | 114 (61) |
| Non-Caucasian | 14 (15) | 85 (21) | 344 (31) | 98 (32) | 13 (25) | 342 (53) | 19 (40) | 72 (39) |
| | GBM (N = 619) | HNSC (N = 530) | KICH (N = 113) | KIRC (N = 538) | KIRP (N = 293) | LAML (N = 200) | LGG (N = 530) | LIHC (N = 442) |
| --- | --- | --- | --- | --- | --- | --- | --- | --- |
| | Glioblastoma Multiforme | Head and Neck Squamous Cell Carcinoma | Kidney Chromophobe | Kidney Renal Clear Cell Carcinoma | Kidney Renal Papillary Cell Carcinoma | Acute Myeloid Leukemia | Brain Lower Grade Glioma | Liver Hepatocellular Carcinoma |
| FADS1 – mean (SD) | -0.0771 (0.9896) | 0.0600 (0.9829) | -0.0412 (0.8899) | 0.0604 (1.0664) | 0.0397 (1.0027) | 0.0521 (1.0100) | -0.0129 (1.0168) | 0.0529 (1.8825) |
| Missing | 453 | 8 | 47 | 4 | 2 | 27 | 0 | 69 |
| Age – median (range) | 59 (10-89) | 61 (19-90) | 50 (17-86) | 61 (26-90) | 61 (28-88) | 57.5 (18-88) | 41 (14-87) | 61 (16-90) |
| Missing | 10 | 1 | 0 | 0 | 4 | 0 | 1 | 64 |
| Race – no. (%) | | | | | | | | |
| Caucasian | 518 (84) | 454 (86) | 95 (84) | 466 (87) | 207 (71) | 181 (90) | 488 (92) | 188 (43) |
| Non-Caucasian | 101 (16) | 76 (14) | 18 (16) | 72 (13) | 86 (29) | 19 (10) | 42 (8) | 254 (57) |
| | LUAD (N = 586) | LUSC (N = 511) | MESO (N = 87) | OV (N = 617) | PAAD (N = 186) | PCPG (N = 184) | PRAD (N = 501) | SARC (N = 265) |
| --- | --- | --- | --- | --- | --- | --- | --- | --- |
| | Lung Adenocarcinoma | Lung Squamous Cell Carcinoma | Mesothelioma | Ovarian Serous Cystadenocarcinoma | Pancreatic Adenocarcinoma | Pheochromocytoma and Paraganglioma | Prostate Adenocarcinoma | Sarcoma |
| FADS1 – mean (SD) | 0.1957 (1.1442) | 0.0896 (1.0880) | 0.1083 (1.1035) | 0.1477 (1.3281) | 0.0015 (0.9982) | -0.1462 (1.0470) | 0.0034 (1.0013) | -0.0480 (1.0056) |
| Missing | 69 | 10 | 0 | 310 | 7 | 0 | 3 | 2 |
| Age – median (range) | 66 (33-88) | 68 (39-90) | 64 (28-81) | 59 (26-89) | 65 (35-88) | 46 (19-83) | 61 (41-78) | 61 (20-90) |
| Missing | 81 | 16 | 0 | 13 | 0 | 0 | 0 | 0 |
| Race – no. (%) | | | | | | | | |
| Caucasian | 394 (67) | 351 (69) | 85 (98) | 514 (83) | 163 (88) | 153 (83) | 147 (29) | 232 (88) |
| Non-Caucasian | 192 (33) | 160 (31) | 2 (2) | 103 (17) | 23 (12) | 31 (17) | 354 (71) | 33 (12) |
| | SKCM (N = 480) | STAD (N = 478) | TGCT (N = 156) | THCA (N = 516) | THYM (N = 124) | UCEC (N = 549) | UCS (N = 57) | UVM (N = 80) |
| --- | --- | --- | --- | --- | --- | --- | --- | --- |
| | Skin Cutaneous Melanoma | Stomach Adenocarcinoma | Testicular Germ Cell Cancer | Thyroid Carcinoma | Thymoma | Uterine Corpus Endometrial Carcinoma | Uterine Carcinosarcoma | Uveal Melanoma |
| FADS1 – mean (SD) | -0.1469 (0.9256) | 0.0534 (1.0393) | -0.5240 (0.8105) | 0.0286 (1.0581) | -0.0130 (1.1131) | 0.0885 (1.0902) | -0.0599 (1.2536) | 0.0210 (0.9684) |
| Missing | 8 | 63 | 0 | 7 | 4 | 372 | 0 | 0 |
| Age – median (range) | 58 (15-90) | 67 (30-90) | 31 (14-67) | 46 (15-89) | 60 (17-84) | 64 (31-90) | 68 (51-90) | 61.5 (22-86) |
| Missing | 9 | 40 | 17 | 1 | 1 | 3 | 0 | 0 |
| Race – no. (%) | | | | | | | | |
| Caucasian | 454 (95) | 278 (58) | 124 (79) | 338 (66) | 103 (83) | 375 (68) | 44 (77) | 55 (69) |
| Non-Caucasian | 26 (5) | 200 (42) | 32 (21) | 178 (34) | 21 (17) | 174 (32) | 13 (23) | 25 (31) |

## Slide 3
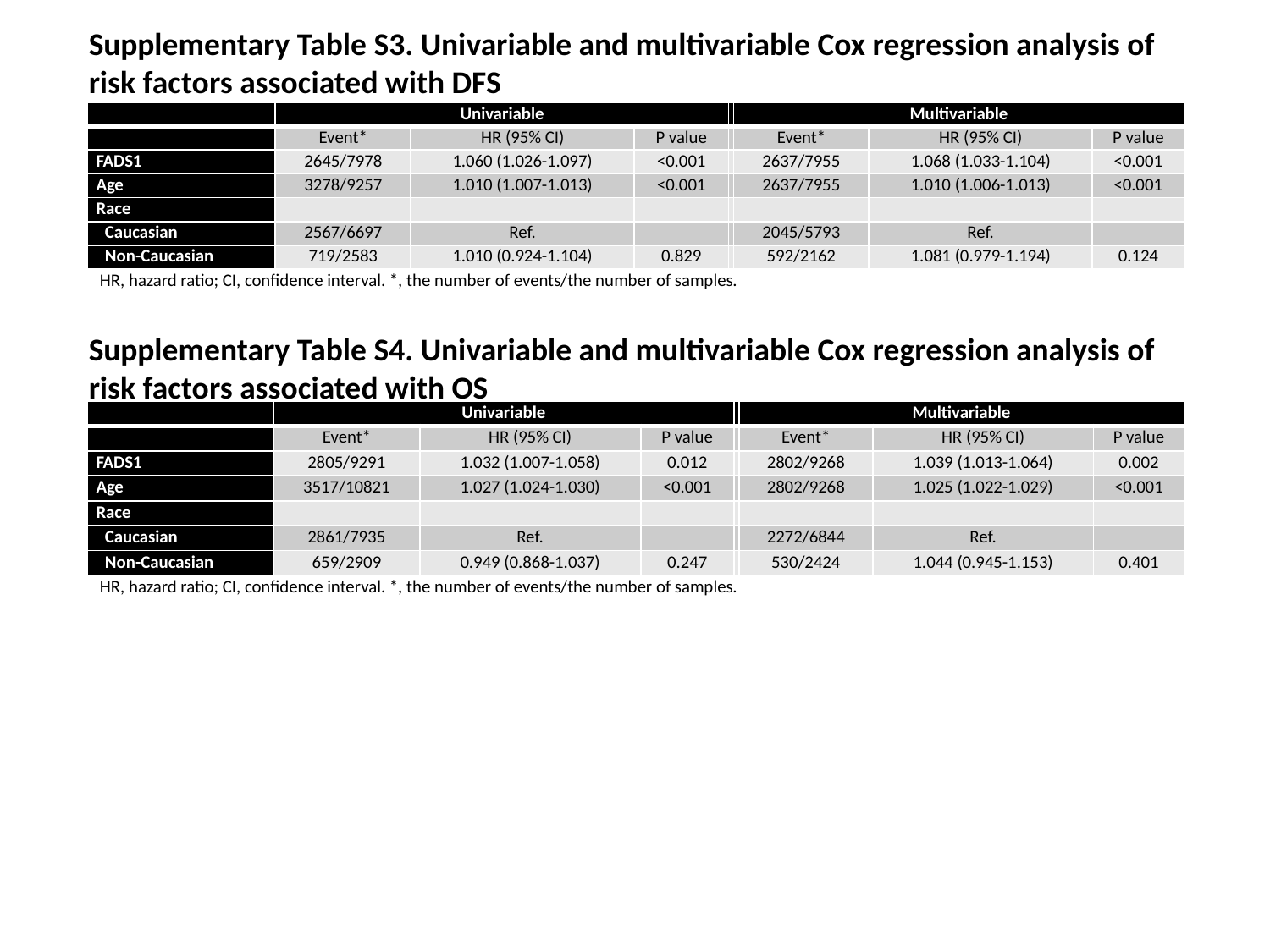

Supplementary Table S3. Univariable and multivariable Cox regression analysis of risk factors associated with DFS
| | Univariable | | | | Multivariable | | |
| --- | --- | --- | --- | --- | --- | --- | --- |
| | Event\* | HR (95% CI) | P value | | Event\* | HR (95% CI) | P value |
| FADS1 | 2645/7978 | 1.060 (1.026-1.097) | <0.001 | | 2637/7955 | 1.068 (1.033-1.104) | <0.001 |
| Age | 3278/9257 | 1.010 (1.007-1.013) | <0.001 | | 2637/7955 | 1.010 (1.006-1.013) | <0.001 |
| Race | | | | | | | |
| Caucasian | 2567/6697 | Ref. | | | 2045/5793 | Ref. | |
| Non-Caucasian | 719/2583 | 1.010 (0.924-1.104) | 0.829 | | 592/2162 | 1.081 (0.979-1.194) | 0.124 |
HR, hazard ratio; CI, confidence interval. *, the number of events/the number of samples.
Supplementary Table S4. Univariable and multivariable Cox regression analysis of risk factors associated with OS
| | Univariable | | | | Multivariable | | |
| --- | --- | --- | --- | --- | --- | --- | --- |
| | Event\* | HR (95% CI) | P value | | Event\* | HR (95% CI) | P value |
| FADS1 | 2805/9291 | 1.032 (1.007-1.058) | 0.012 | | 2802/9268 | 1.039 (1.013-1.064) | 0.002 |
| Age | 3517/10821 | 1.027 (1.024-1.030) | <0.001 | | 2802/9268 | 1.025 (1.022-1.029) | <0.001 |
| Race | | | | | | | |
| Caucasian | 2861/7935 | Ref. | | | 2272/6844 | Ref. | |
| Non-Caucasian | 659/2909 | 0.949 (0.868-1.037) | 0.247 | | 530/2424 | 1.044 (0.945-1.153) | 0.401 |
HR, hazard ratio; CI, confidence interval. *, the number of events/the number of samples.

## Slide 4
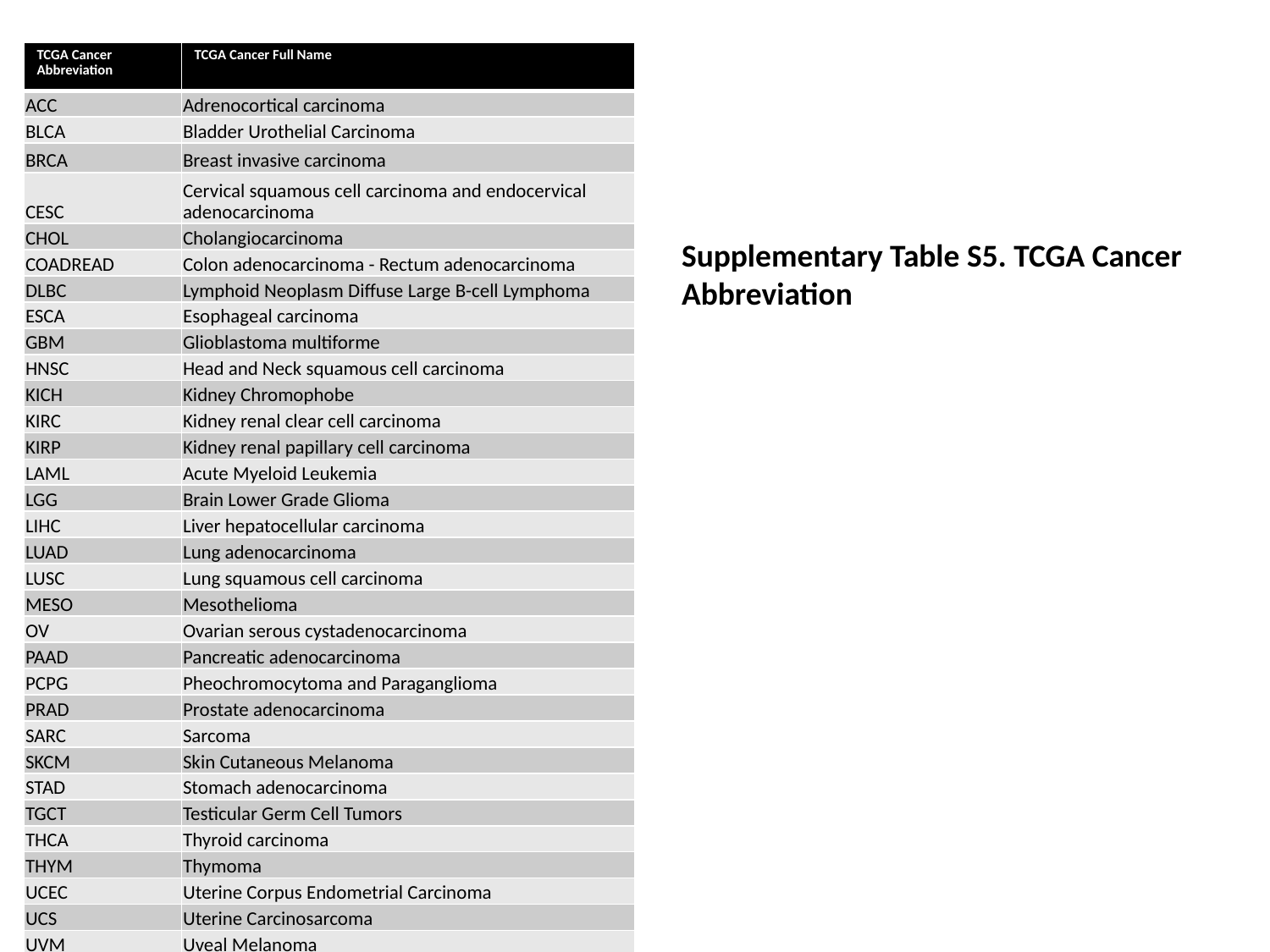

| TCGA Cancer Abbreviation | TCGA Cancer Full Name |
| --- | --- |
| ACC | Adrenocortical carcinoma |
| BLCA | Bladder Urothelial Carcinoma |
| BRCA | Breast invasive carcinoma |
| CESC | Cervical squamous cell carcinoma and endocervical adenocarcinoma |
| CHOL | Cholangiocarcinoma |
| COADREAD | Colon adenocarcinoma - Rectum adenocarcinoma |
| DLBC | Lymphoid Neoplasm Diffuse Large B-cell Lymphoma |
| ESCA | Esophageal carcinoma |
| GBM | Glioblastoma multiforme |
| HNSC | Head and Neck squamous cell carcinoma |
| KICH | Kidney Chromophobe |
| KIRC | Kidney renal clear cell carcinoma |
| KIRP | Kidney renal papillary cell carcinoma |
| LAML | Acute Myeloid Leukemia |
| LGG | Brain Lower Grade Glioma |
| LIHC | Liver hepatocellular carcinoma |
| LUAD | Lung adenocarcinoma |
| LUSC | Lung squamous cell carcinoma |
| MESO | Mesothelioma |
| OV | Ovarian serous cystadenocarcinoma |
| PAAD | Pancreatic adenocarcinoma |
| PCPG | Pheochromocytoma and Paraganglioma |
| PRAD | Prostate adenocarcinoma |
| SARC | Sarcoma |
| SKCM | Skin Cutaneous Melanoma |
| STAD | Stomach adenocarcinoma |
| TGCT | Testicular Germ Cell Tumors |
| THCA | Thyroid carcinoma |
| THYM | Thymoma |
| UCEC | Uterine Corpus Endometrial Carcinoma |
| UCS | Uterine Carcinosarcoma |
| UVM | Uveal Melanoma |
Supplementary Table S5. TCGA Cancer Abbreviation

## Slide 5
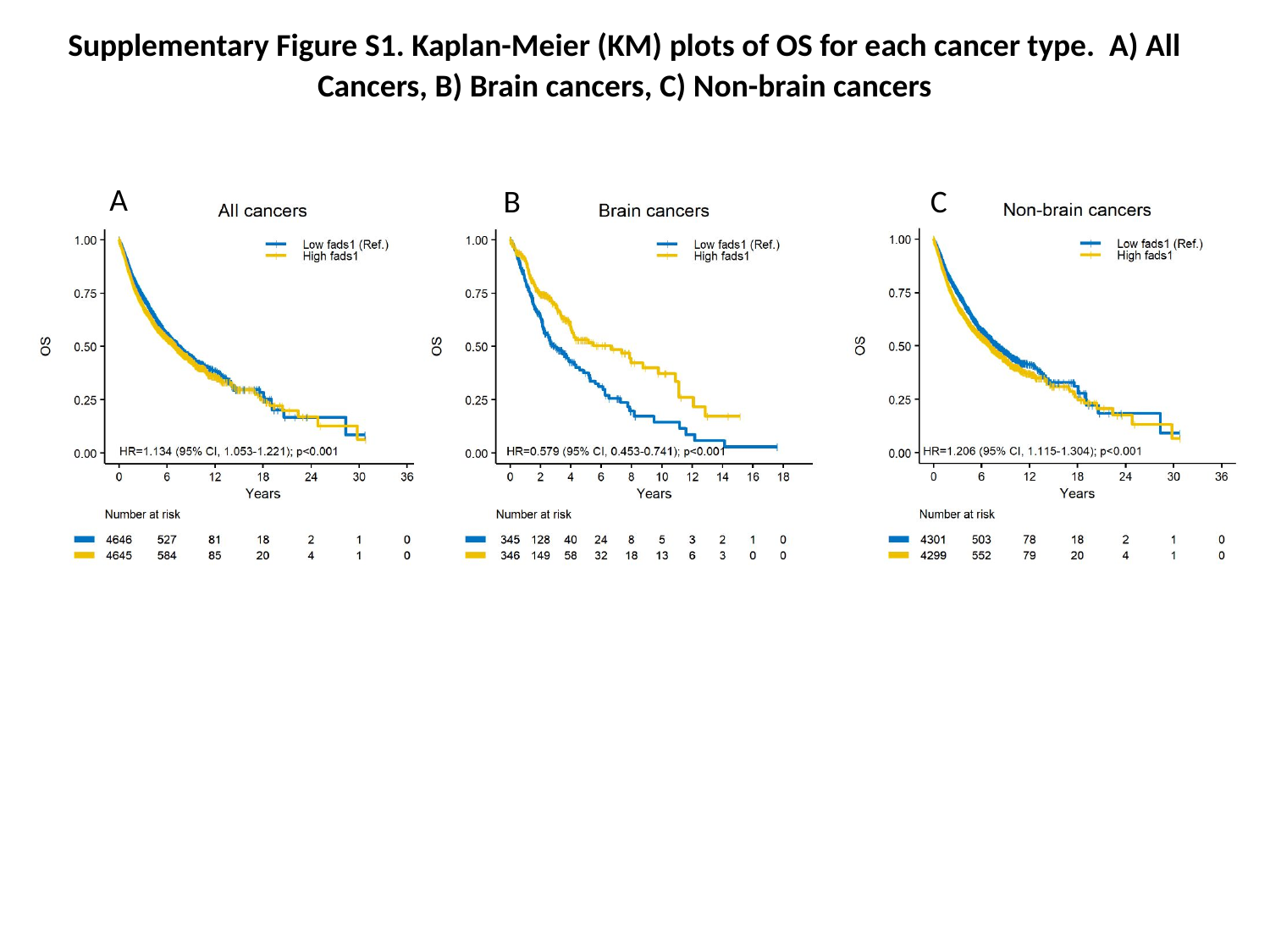

Supplementary Figure S1. Kaplan-Meier (KM) plots of OS for each cancer type. A) All Cancers, B) Brain cancers, C) Non-brain cancers
A
B
C

## Slide 6
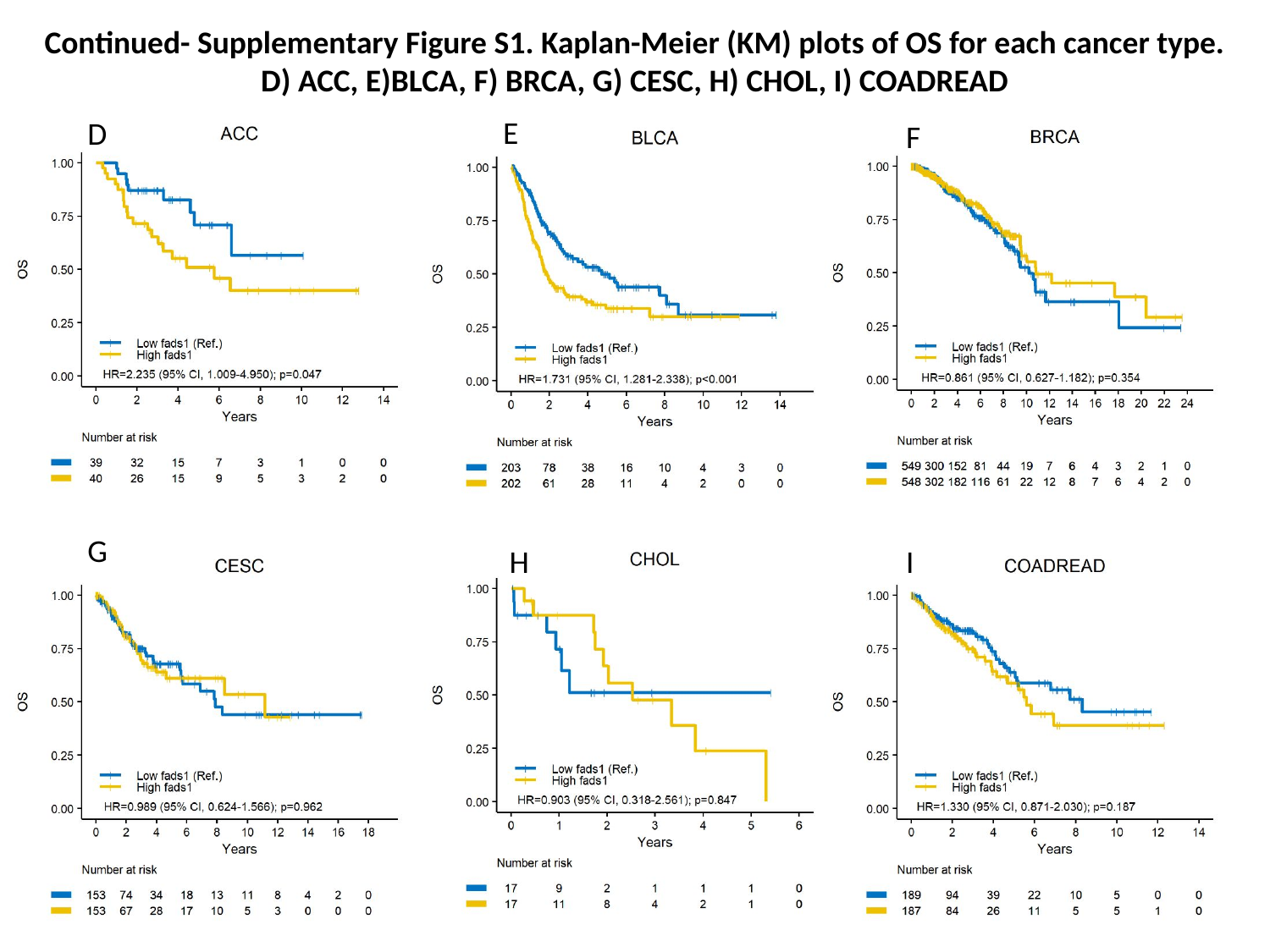

Continued- Supplementary Figure S1. Kaplan-Meier (KM) plots of OS for each cancer type. D) ACC, E)BLCA, F) BRCA, G) CESC, H) CHOL, I) COADREAD
E
D
F
G
H
I

## Slide 7
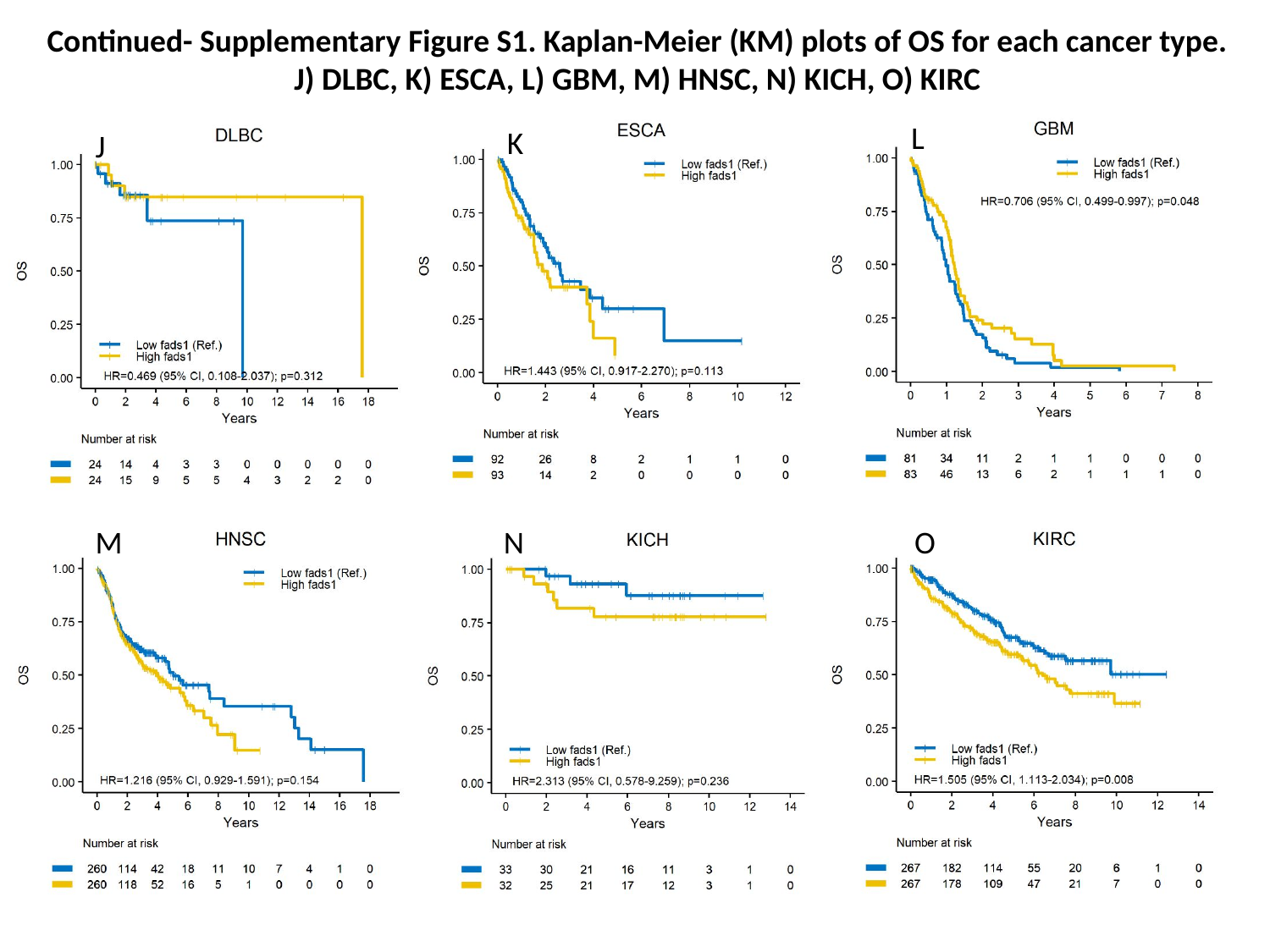

Continued- Supplementary Figure S1. Kaplan-Meier (KM) plots of OS for each cancer type. J) DLBC, K) ESCA, L) GBM, M) HNSC, N) KICH, O) KIRC
L
K
J
M
N
O

## Slide 8
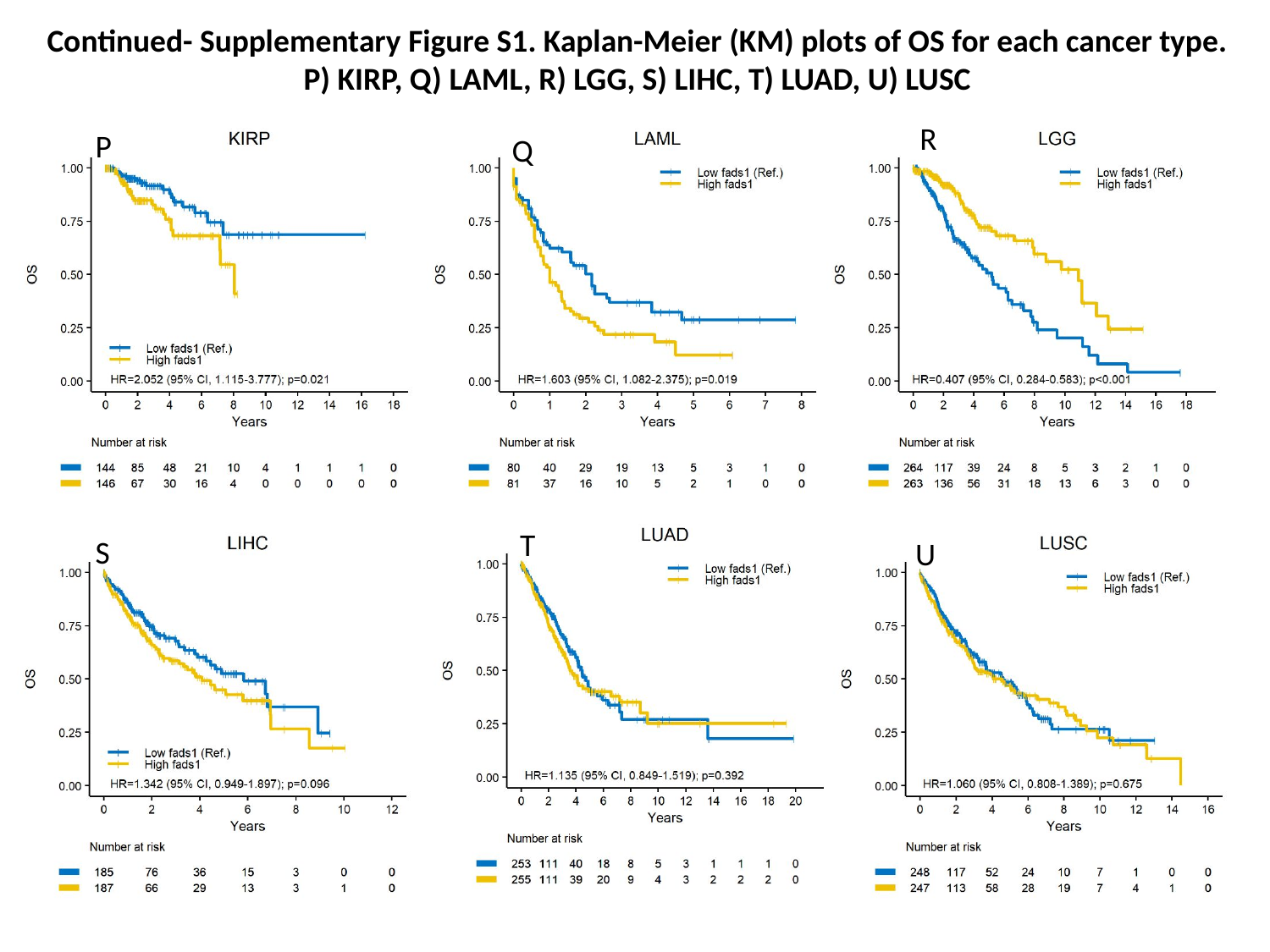

Continued- Supplementary Figure S1. Kaplan-Meier (KM) plots of OS for each cancer type. P) KIRP, Q) LAML, R) LGG, S) LIHC, T) LUAD, U) LUSC
R
P
Q
T
S
U

## Slide 9
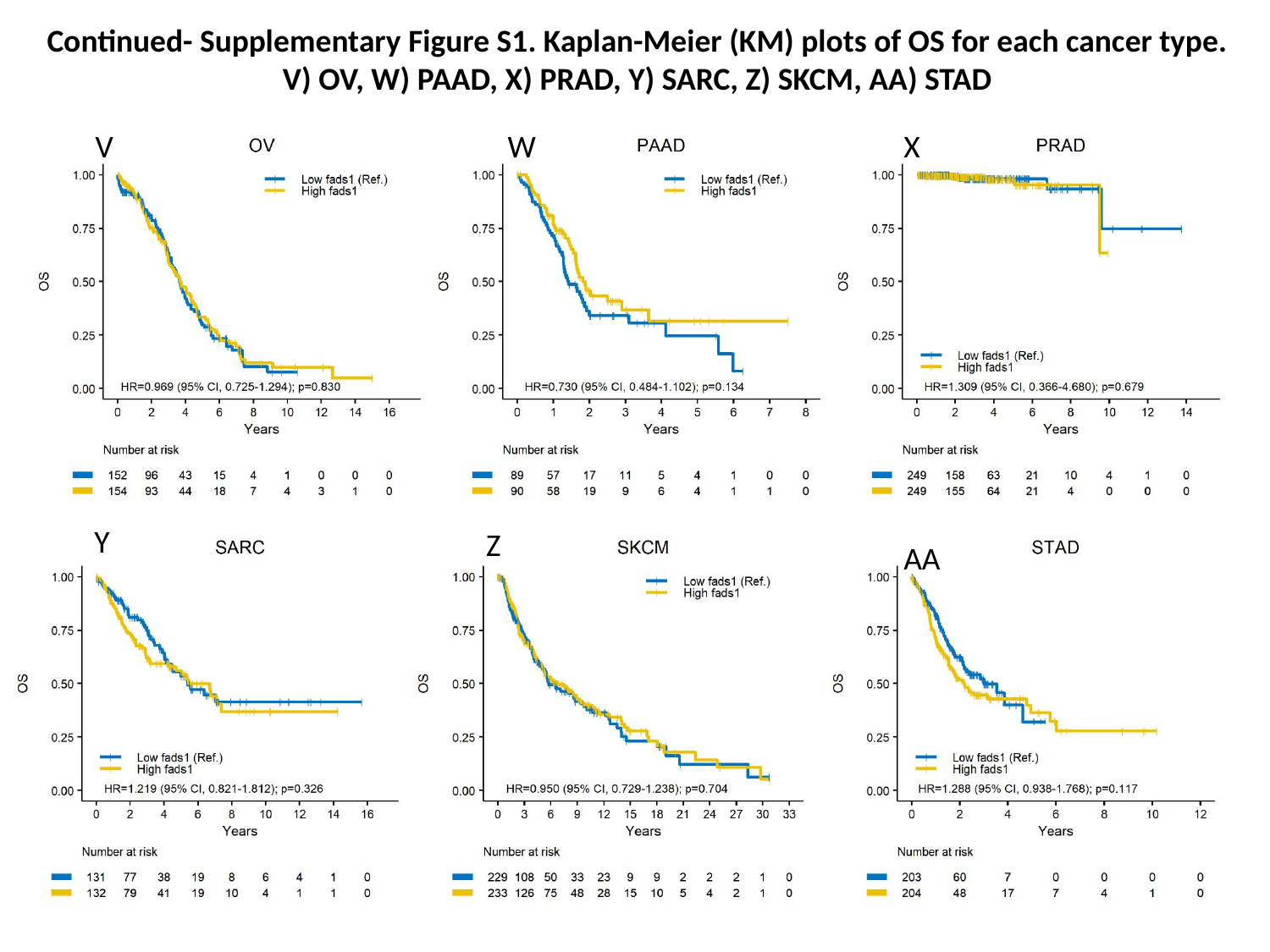

Continued- Supplementary Figure S1. Kaplan-Meier (KM) plots of OS for each cancer type. V) OV, W) PAAD, X) PRAD, Y) SARC, Z) SKCM, AA) STAD
V
W
X
Y
Z
AA

## Slide 10
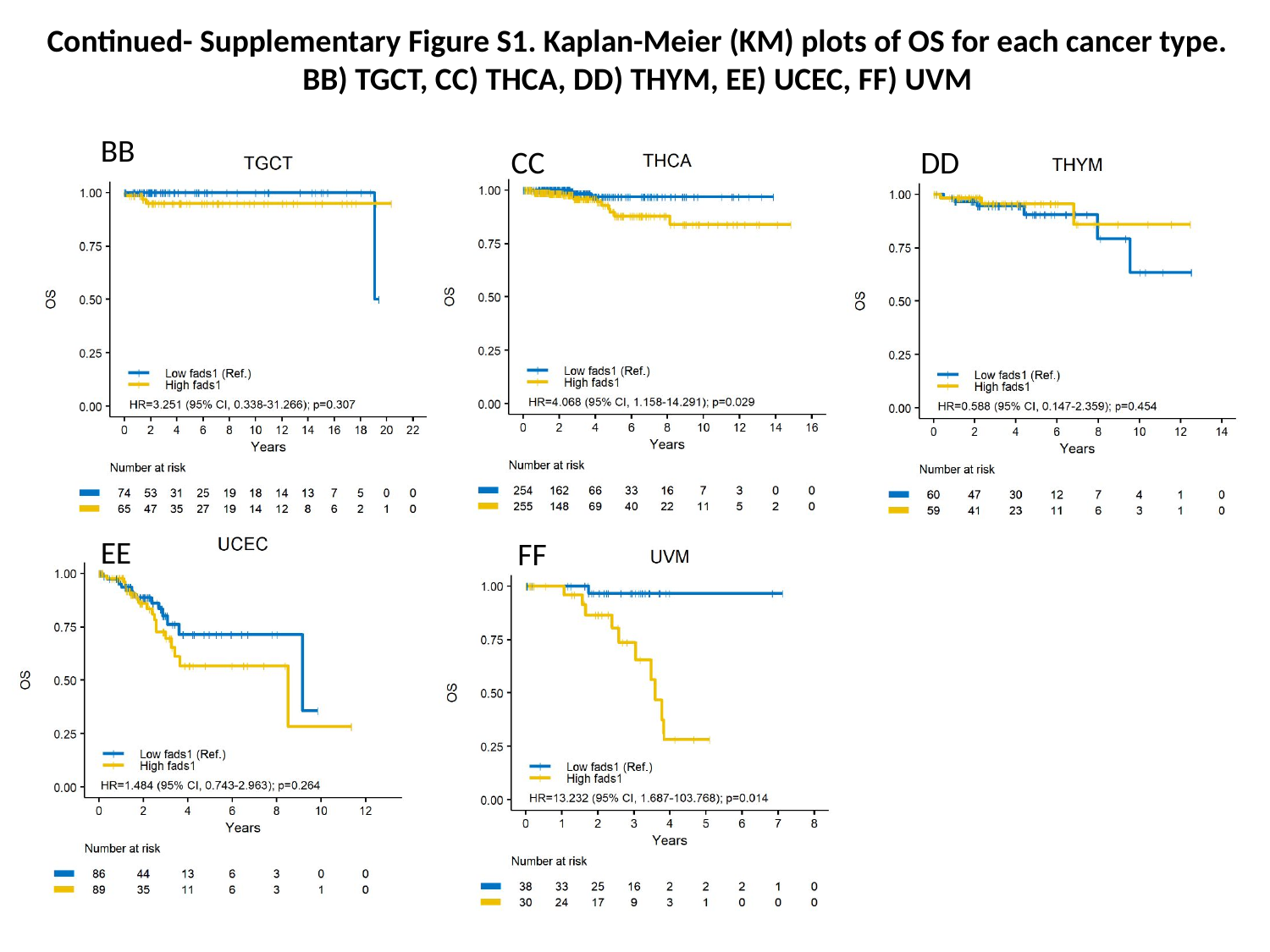

Continued- Supplementary Figure S1. Kaplan-Meier (KM) plots of OS for each cancer type. BB) TGCT, CC) THCA, DD) THYM, EE) UCEC, FF) UVM
BB
CC
DD
EE
FF

## Slide 11
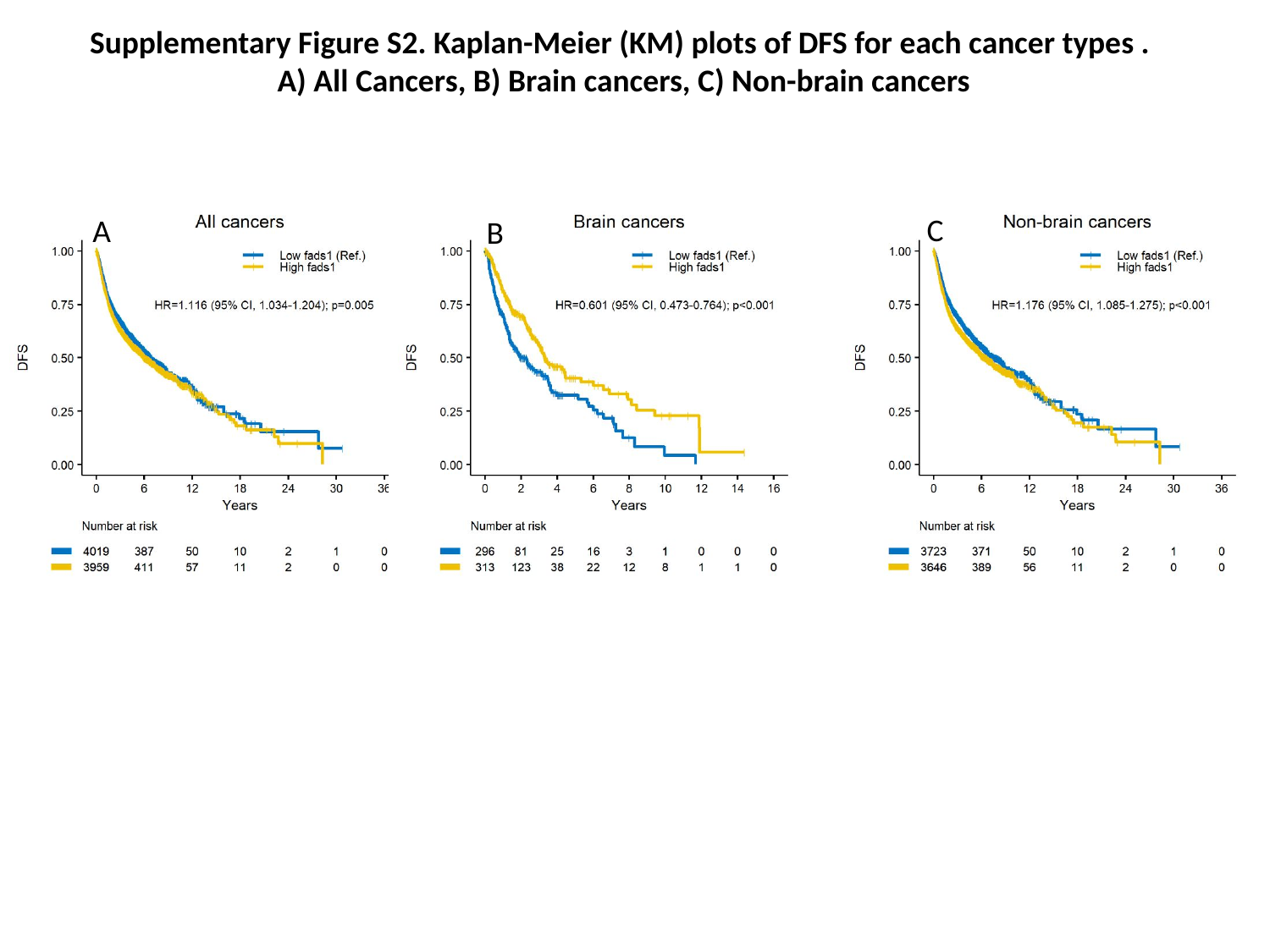

Supplementary Figure S2. Kaplan-Meier (KM) plots of DFS for each cancer types .
A) All Cancers, B) Brain cancers, C) Non-brain cancers
C
A
B

## Slide 12
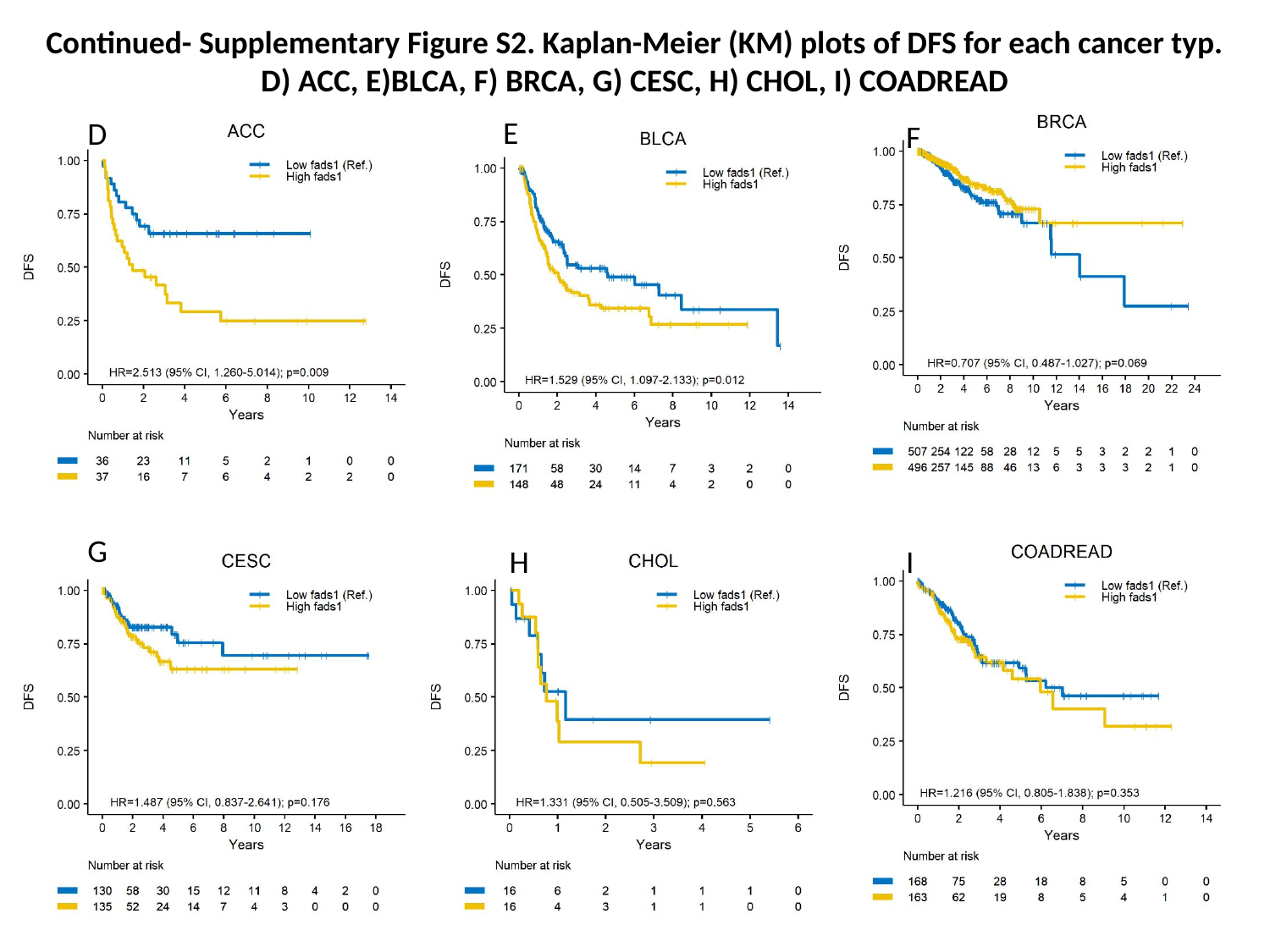

Continued- Supplementary Figure S2. Kaplan-Meier (KM) plots of DFS for each cancer typ. D) ACC, E)BLCA, F) BRCA, G) CESC, H) CHOL, I) COADREAD
E
D
F
G
H
I

## Slide 13
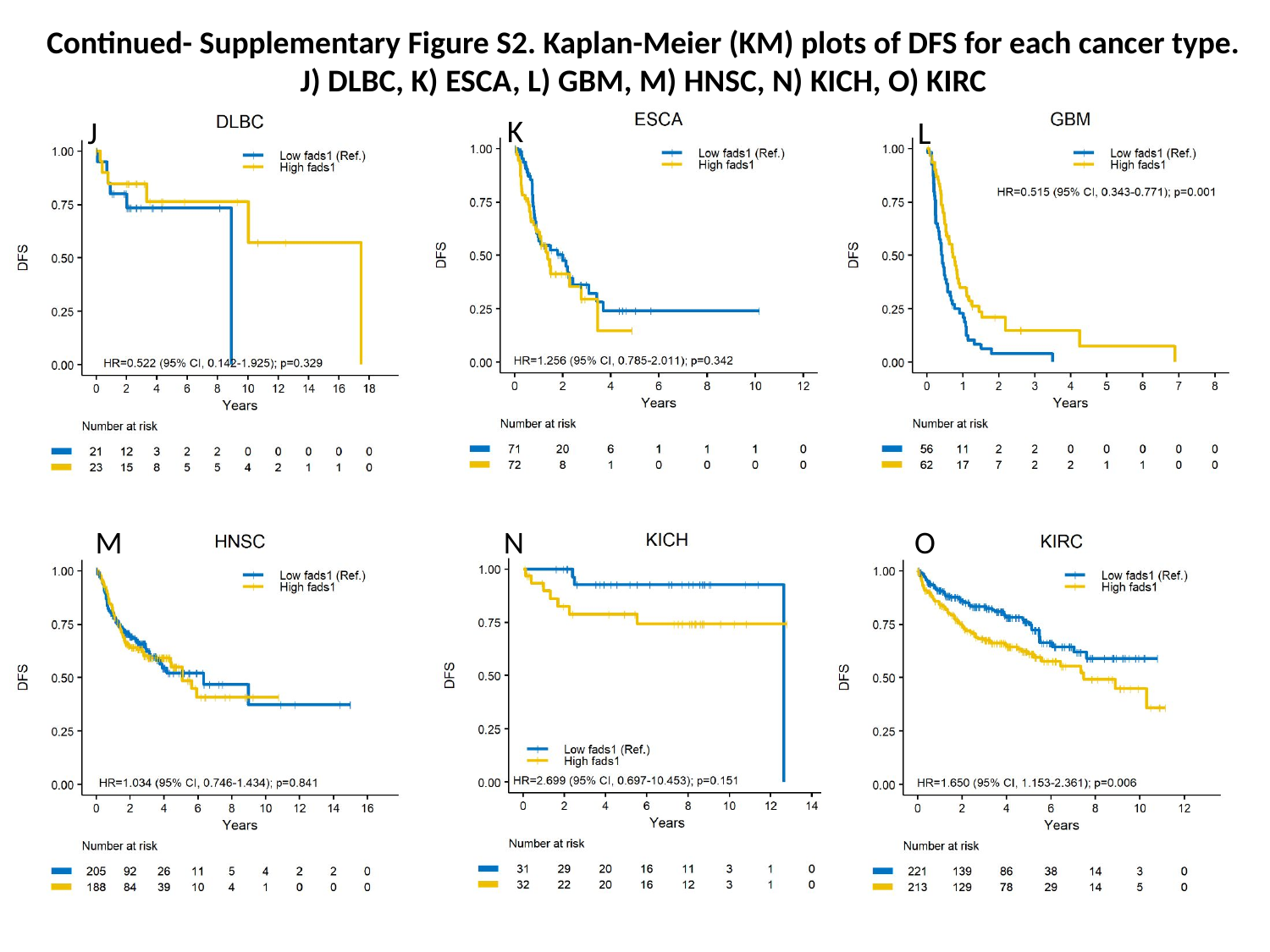

Continued- Supplementary Figure S2. Kaplan-Meier (KM) plots of DFS for each cancer type.
J) DLBC, K) ESCA, L) GBM, M) HNSC, N) KICH, O) KIRC
K
L
J
M
N
O

## Slide 14
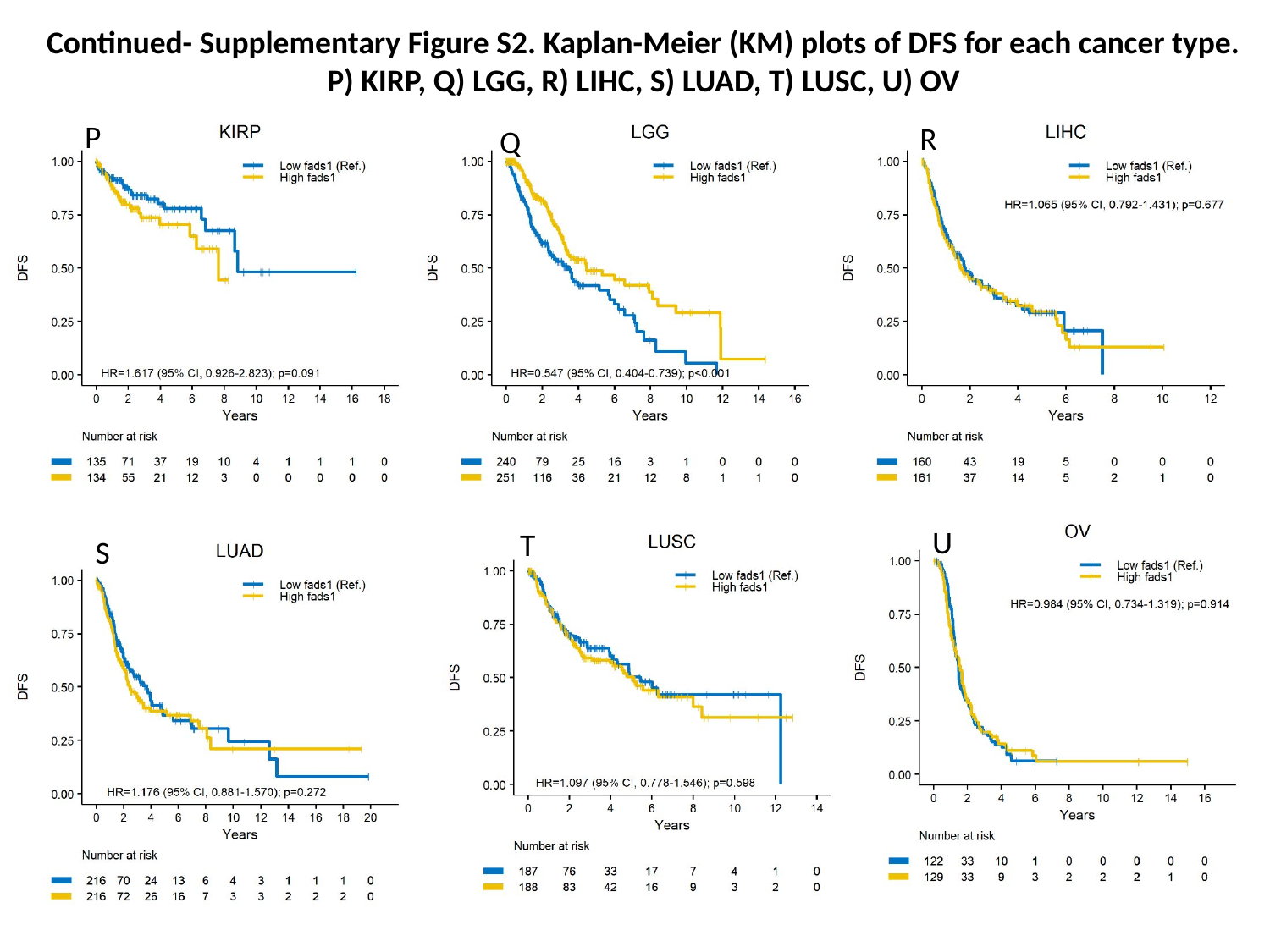

Continued- Supplementary Figure S2. Kaplan-Meier (KM) plots of DFS for each cancer type.
P) KIRP, Q) LGG, R) LIHC, S) LUAD, T) LUSC, U) OV
P
R
Q
U
T
S

## Slide 15
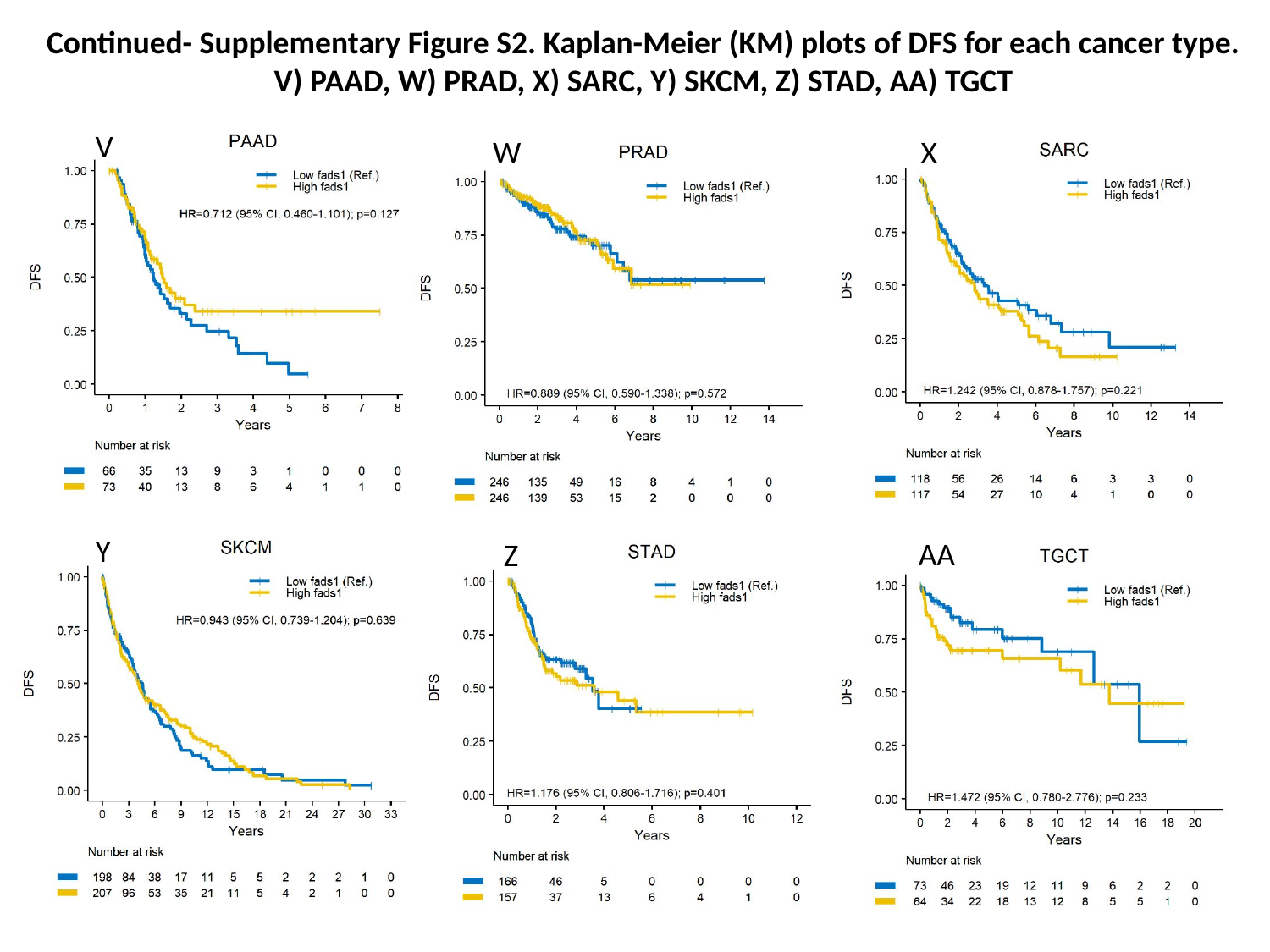

Continued- Supplementary Figure S2. Kaplan-Meier (KM) plots of DFS for each cancer type.
V) PAAD, W) PRAD, X) SARC, Y) SKCM, Z) STAD, AA) TGCT
V
W
X
Y
AA
Z

## Slide 16
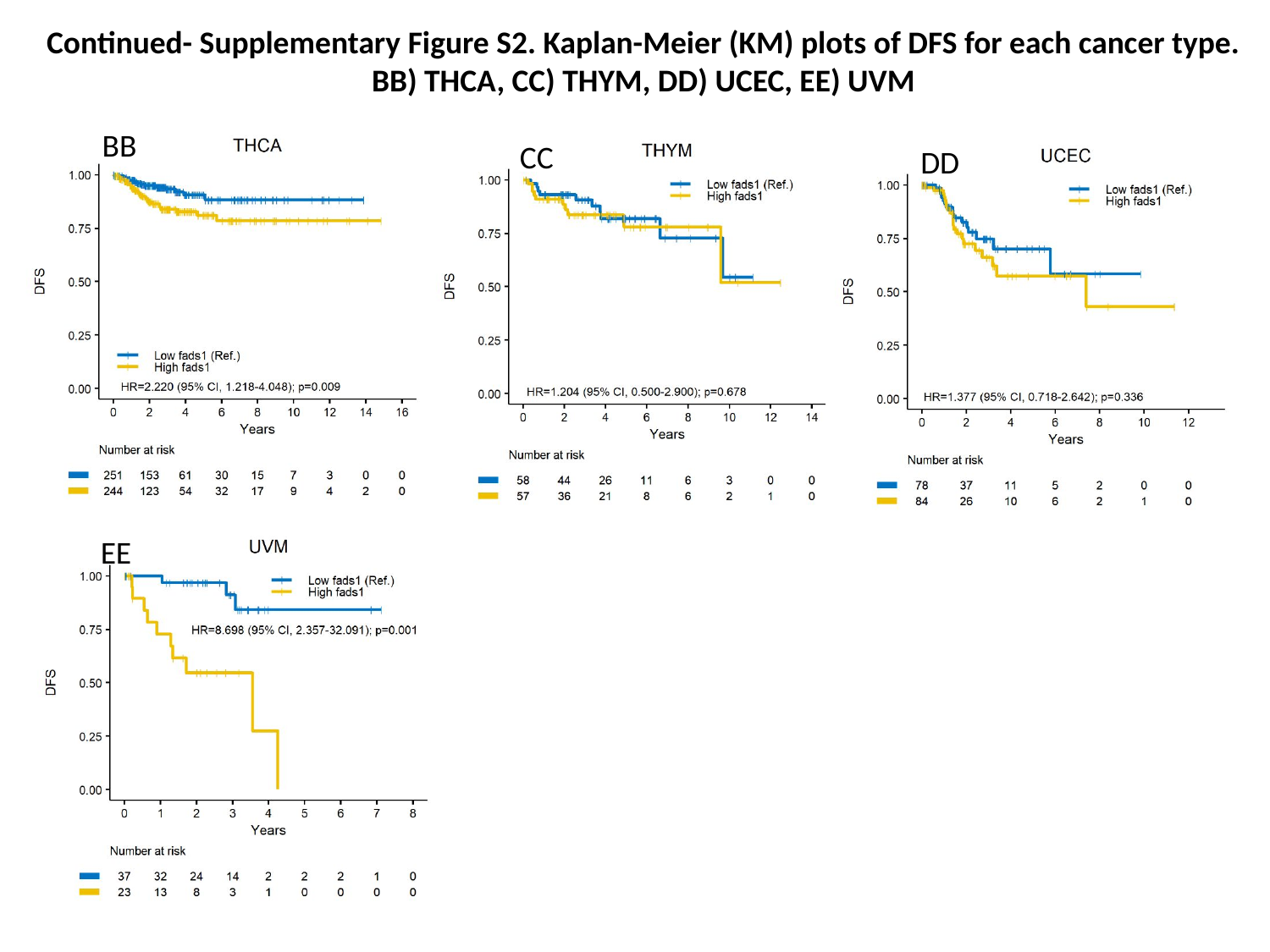

Continued- Supplementary Figure S2. Kaplan-Meier (KM) plots of DFS for each cancer type.
BB) THCA, CC) THYM, DD) UCEC, EE) UVM
BB
CC
DD
EE

## Slide 17
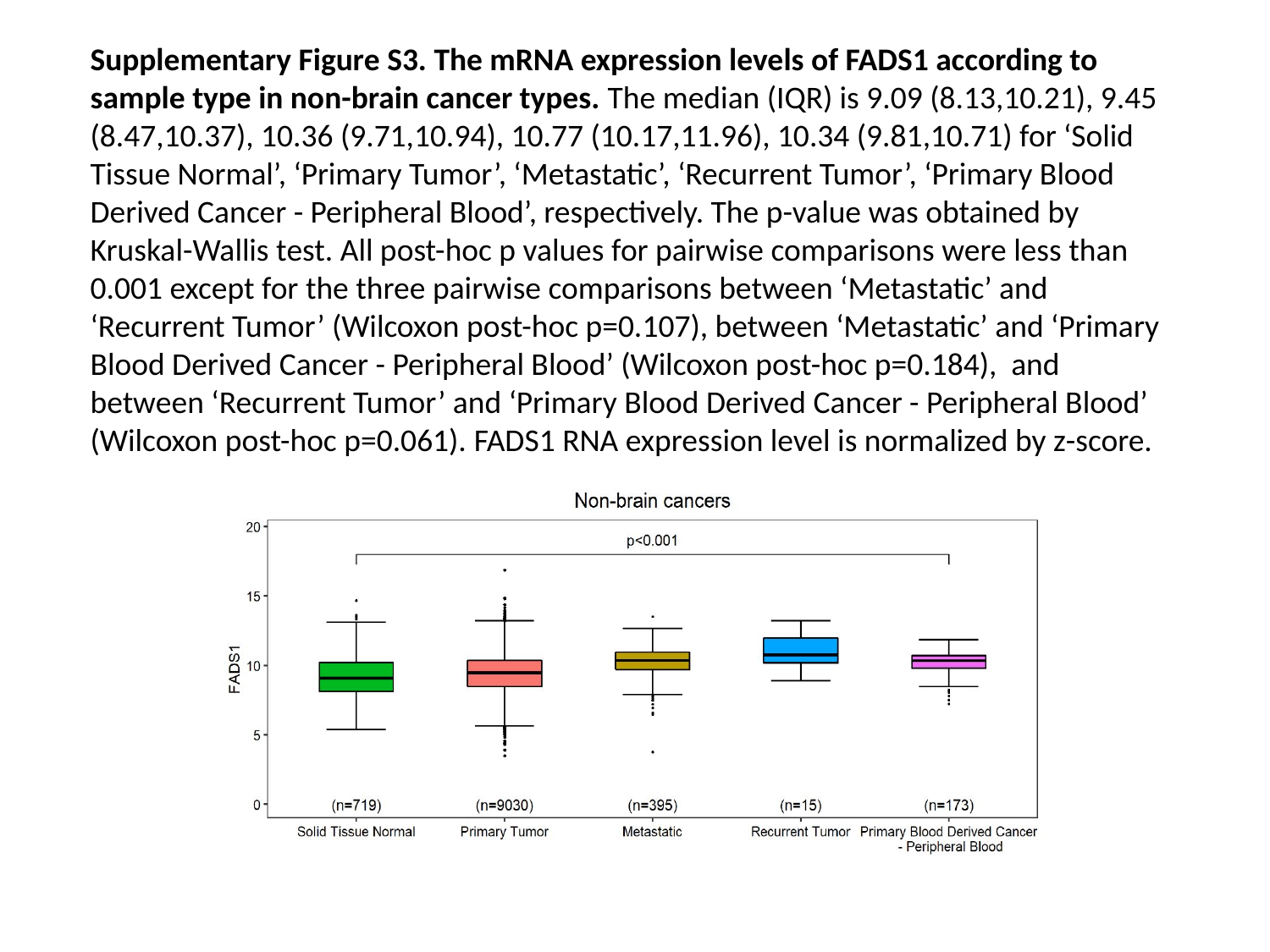

Supplementary Figure S3. The mRNA expression levels of FADS1 according to sample type in non-brain cancer types. The median (IQR) is 9.09 (8.13,10.21), 9.45 (8.47,10.37), 10.36 (9.71,10.94), 10.77 (10.17,11.96), 10.34 (9.81,10.71) for ‘Solid Tissue Normal’, ‘Primary Tumor’, ‘Metastatic’, ‘Recurrent Tumor’, ‘Primary Blood Derived Cancer - Peripheral Blood’, respectively. The p-value was obtained by Kruskal-Wallis test. All post-hoc p values for pairwise comparisons were less than 0.001 except for the three pairwise comparisons between ‘Metastatic’ and ‘Recurrent Tumor’ (Wilcoxon post-hoc p=0.107), between ‘Metastatic’ and ‘Primary Blood Derived Cancer - Peripheral Blood’ (Wilcoxon post-hoc p=0.184), and between ‘Recurrent Tumor’ and ‘Primary Blood Derived Cancer - Peripheral Blood’ (Wilcoxon post-hoc p=0.061). FADS1 RNA expression level is normalized by z-score.

## Slide 18
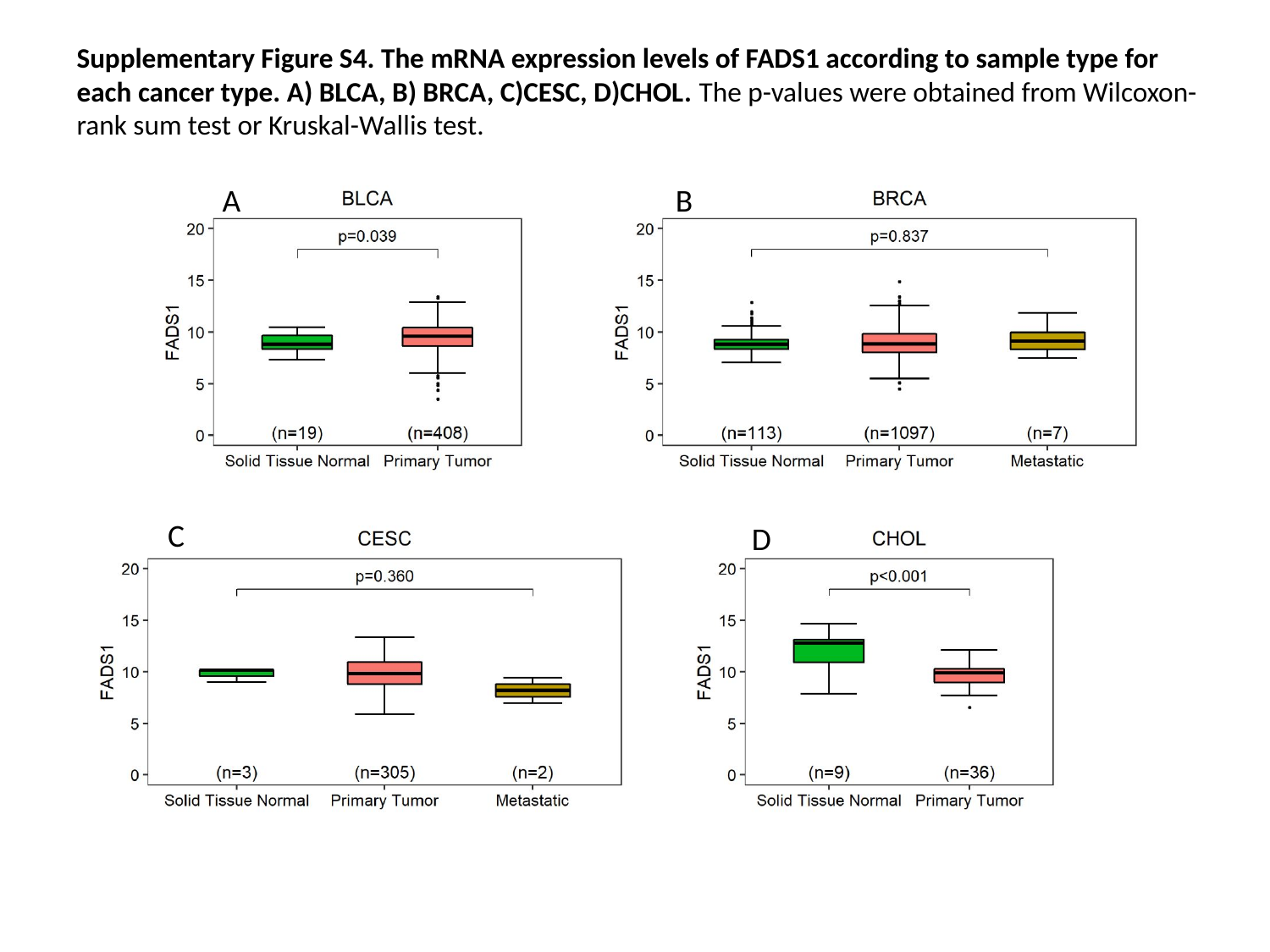

Supplementary Figure S4. The mRNA expression levels of FADS1 according to sample type for each cancer type. A) BLCA, B) BRCA, C)CESC, D)CHOL. The p-values were obtained from Wilcoxon-rank sum test or Kruskal-Wallis test.
A
B
C
D

## Slide 19
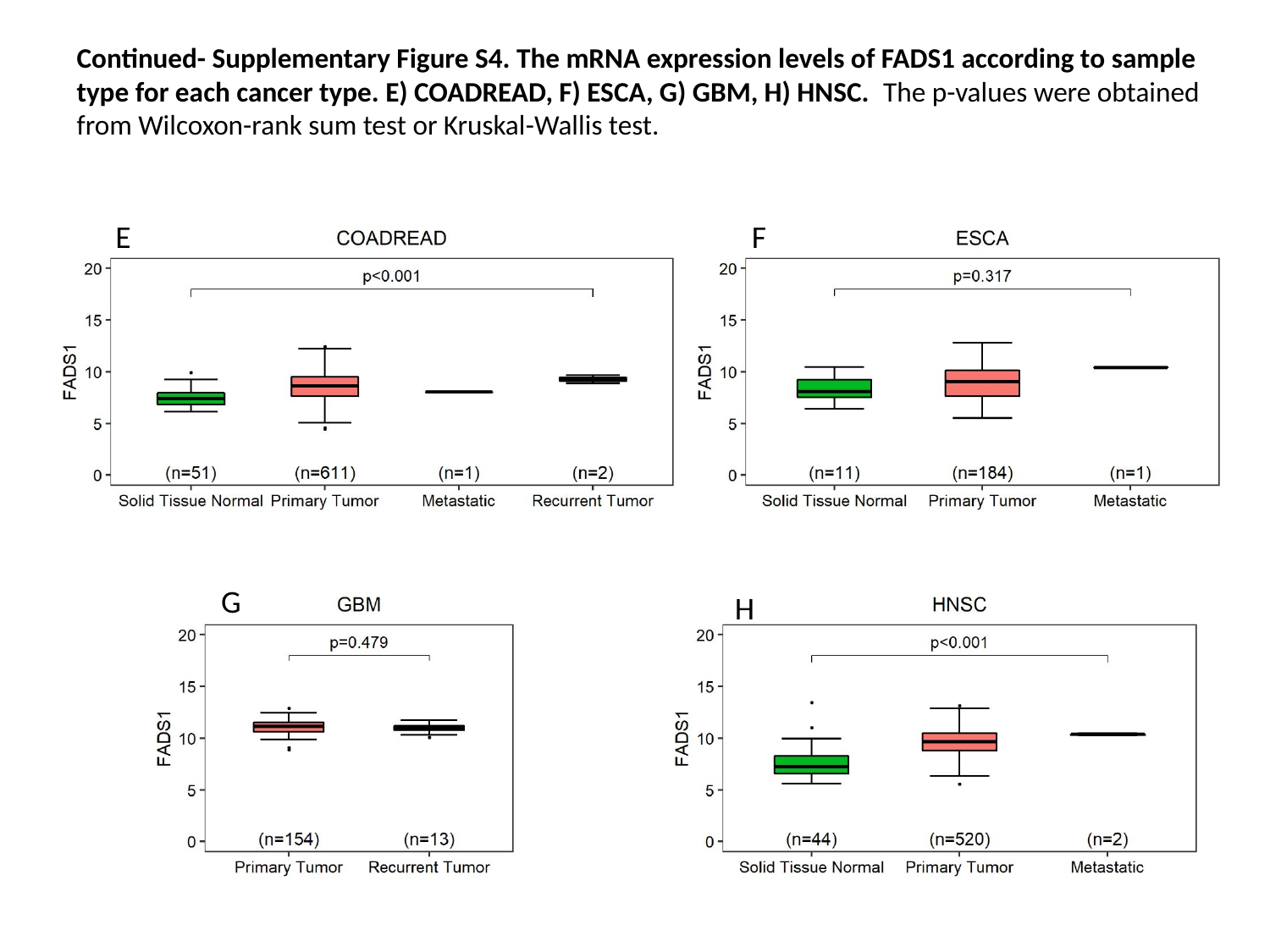

Continued- Supplementary Figure S4. The mRNA expression levels of FADS1 according to sample type for each cancer type. E) COADREAD, F) ESCA, G) GBM, H) HNSC. The p-values were obtained from Wilcoxon-rank sum test or Kruskal-Wallis test.
E
F
G
H

## Slide 20
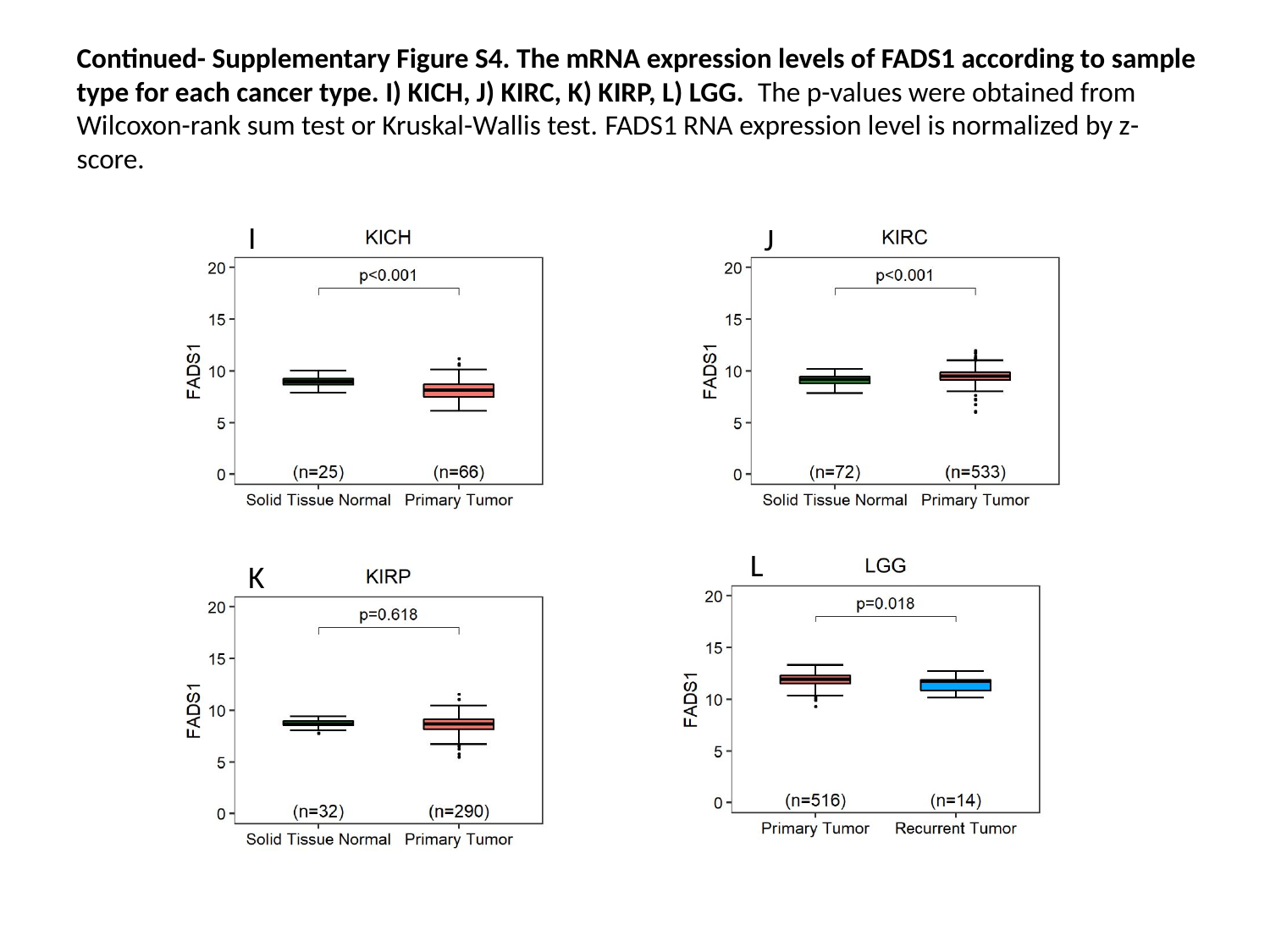

Continued- Supplementary Figure S4. The mRNA expression levels of FADS1 according to sample type for each cancer type. I) KICH, J) KIRC, K) KIRP, L) LGG. The p-values were obtained from Wilcoxon-rank sum test or Kruskal-Wallis test. FADS1 RNA expression level is normalized by z-score.
I
J
L
K

## Slide 21
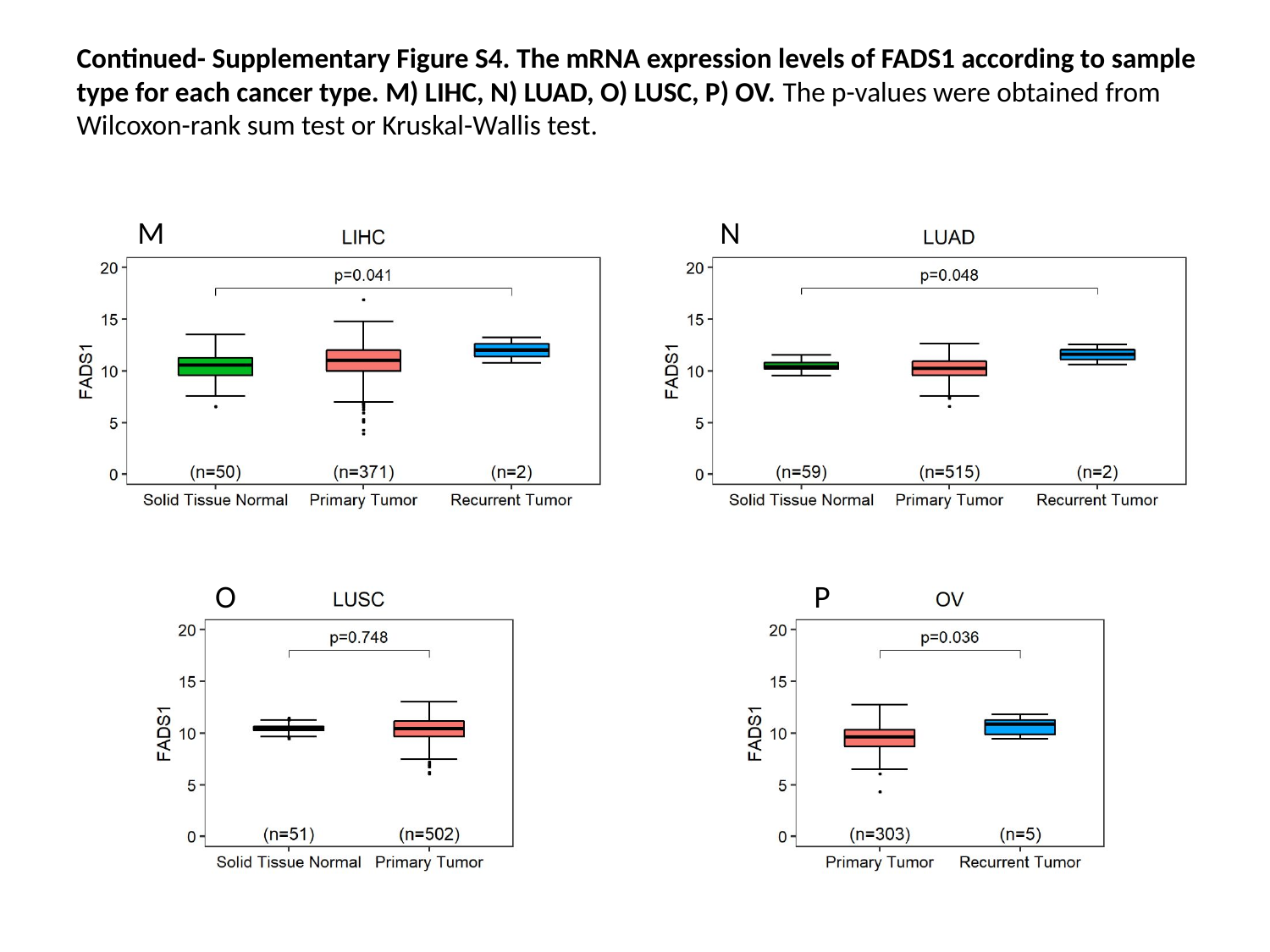

Continued- Supplementary Figure S4. The mRNA expression levels of FADS1 according to sample type for each cancer type. M) LIHC, N) LUAD, O) LUSC, P) OV. The p-values were obtained from Wilcoxon-rank sum test or Kruskal-Wallis test.
M
N
O
P

## Slide 22
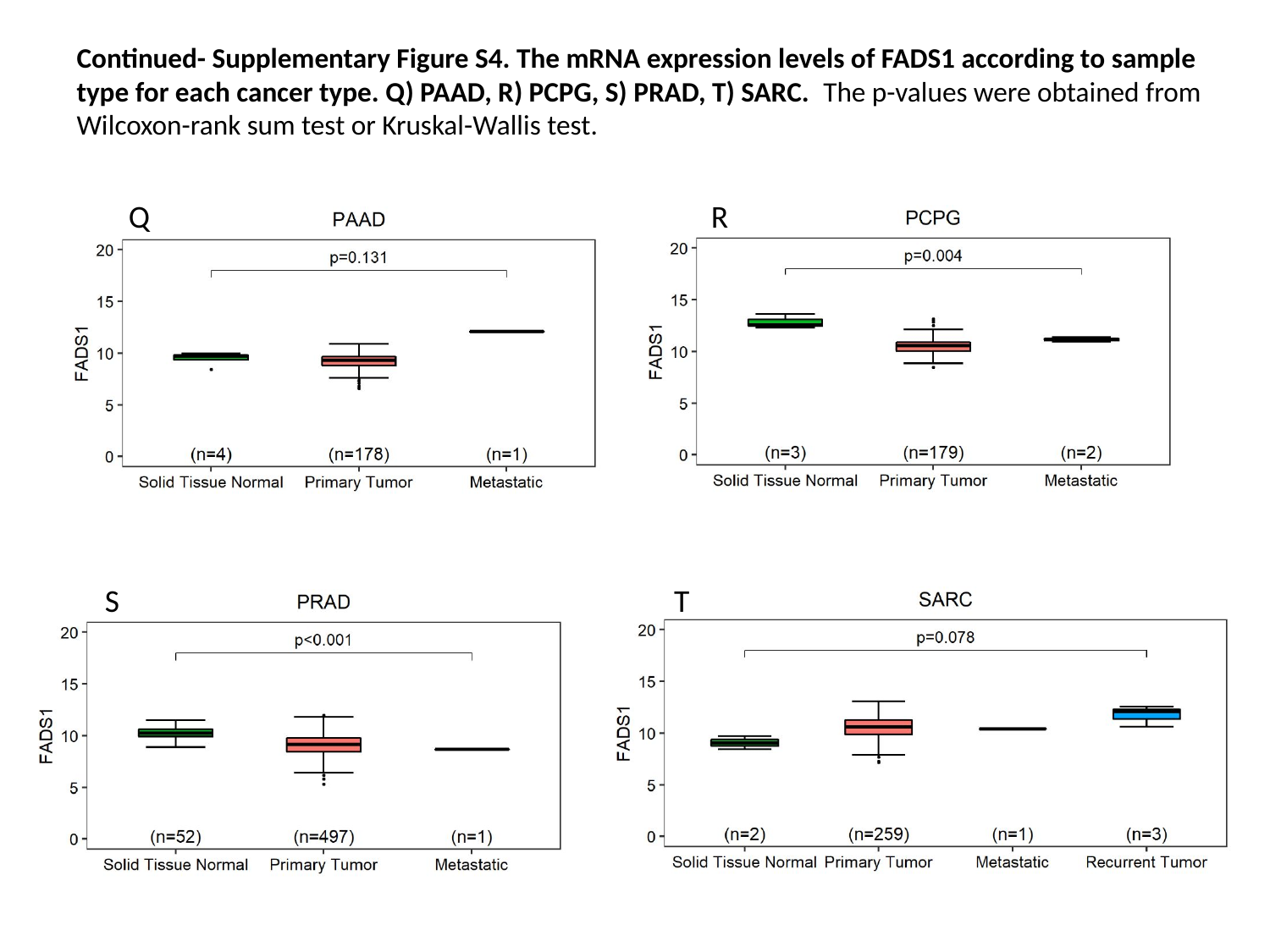

Continued- Supplementary Figure S4. The mRNA expression levels of FADS1 according to sample type for each cancer type. Q) PAAD, R) PCPG, S) PRAD, T) SARC. The p-values were obtained from Wilcoxon-rank sum test or Kruskal-Wallis test.
Q
R
T
S

## Slide 23
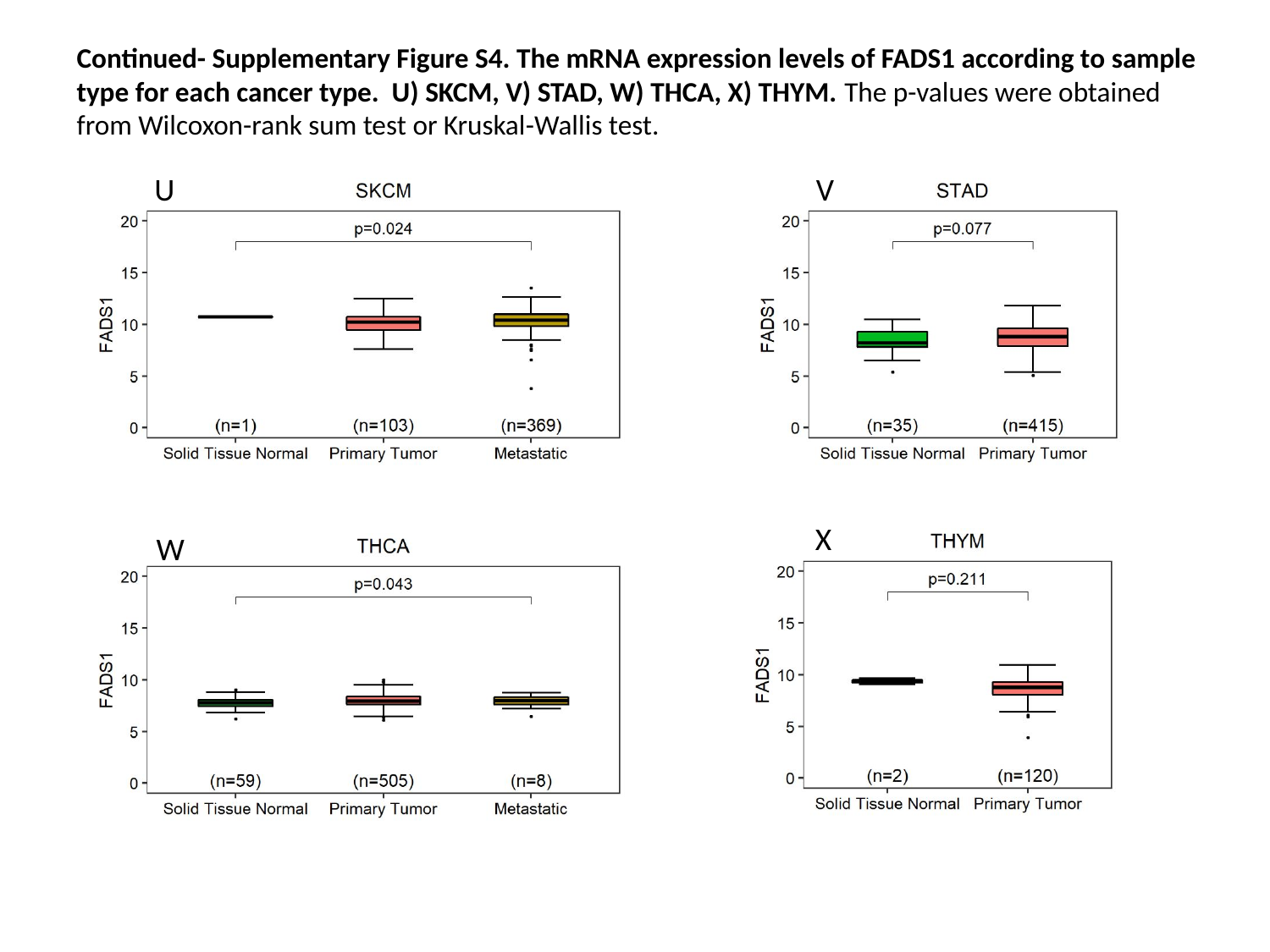

Continued- Supplementary Figure S4. The mRNA expression levels of FADS1 according to sample type for each cancer type. U) SKCM, V) STAD, W) THCA, X) THYM. The p-values were obtained from Wilcoxon-rank sum test or Kruskal-Wallis test.
U
V
X
W

## Slide 24
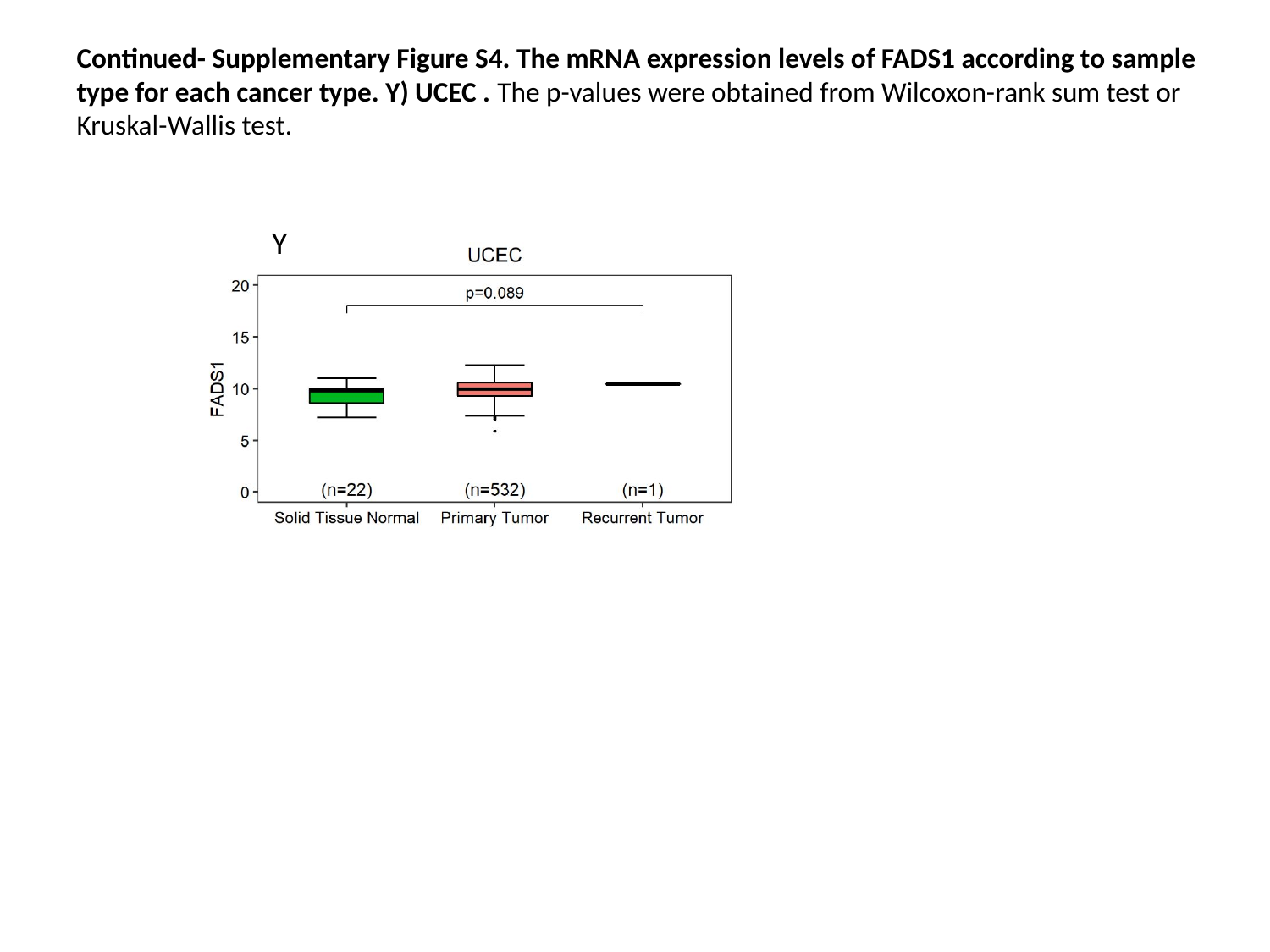

Continued- Supplementary Figure S4. The mRNA expression levels of FADS1 according to sample type for each cancer type. Y) UCEC . The p-values were obtained from Wilcoxon-rank sum test or Kruskal-Wallis test.
Y

## Slide 25
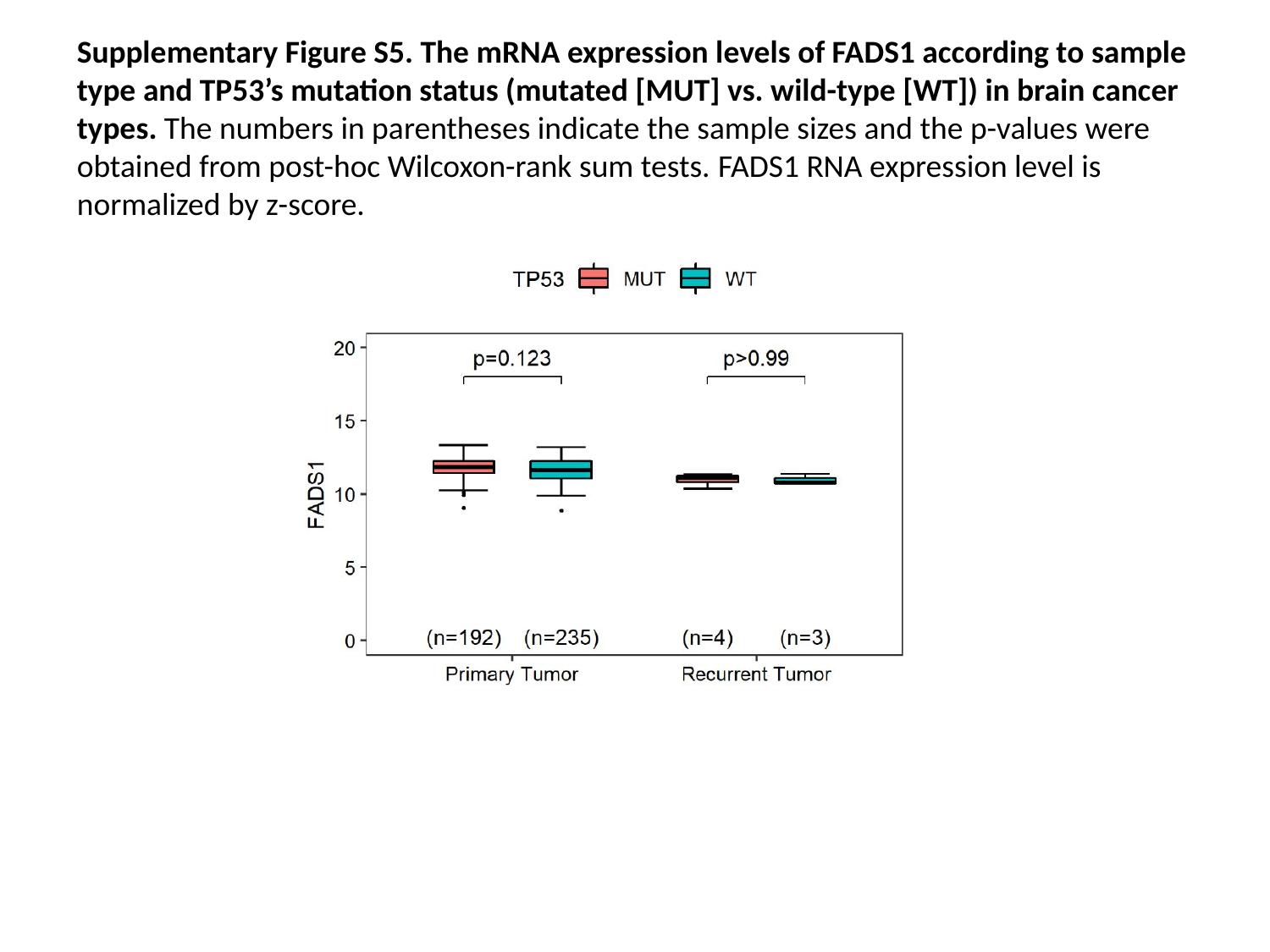

Supplementary Figure S5. The mRNA expression levels of FADS1 according to sample type and TP53’s mutation status (mutated [MUT] vs. wild-type [WT]) in brain cancer types. The numbers in parentheses indicate the sample sizes and the p-values were obtained from post-hoc Wilcoxon-rank sum tests. FADS1 RNA expression level is normalized by z-score.

## Slide 26
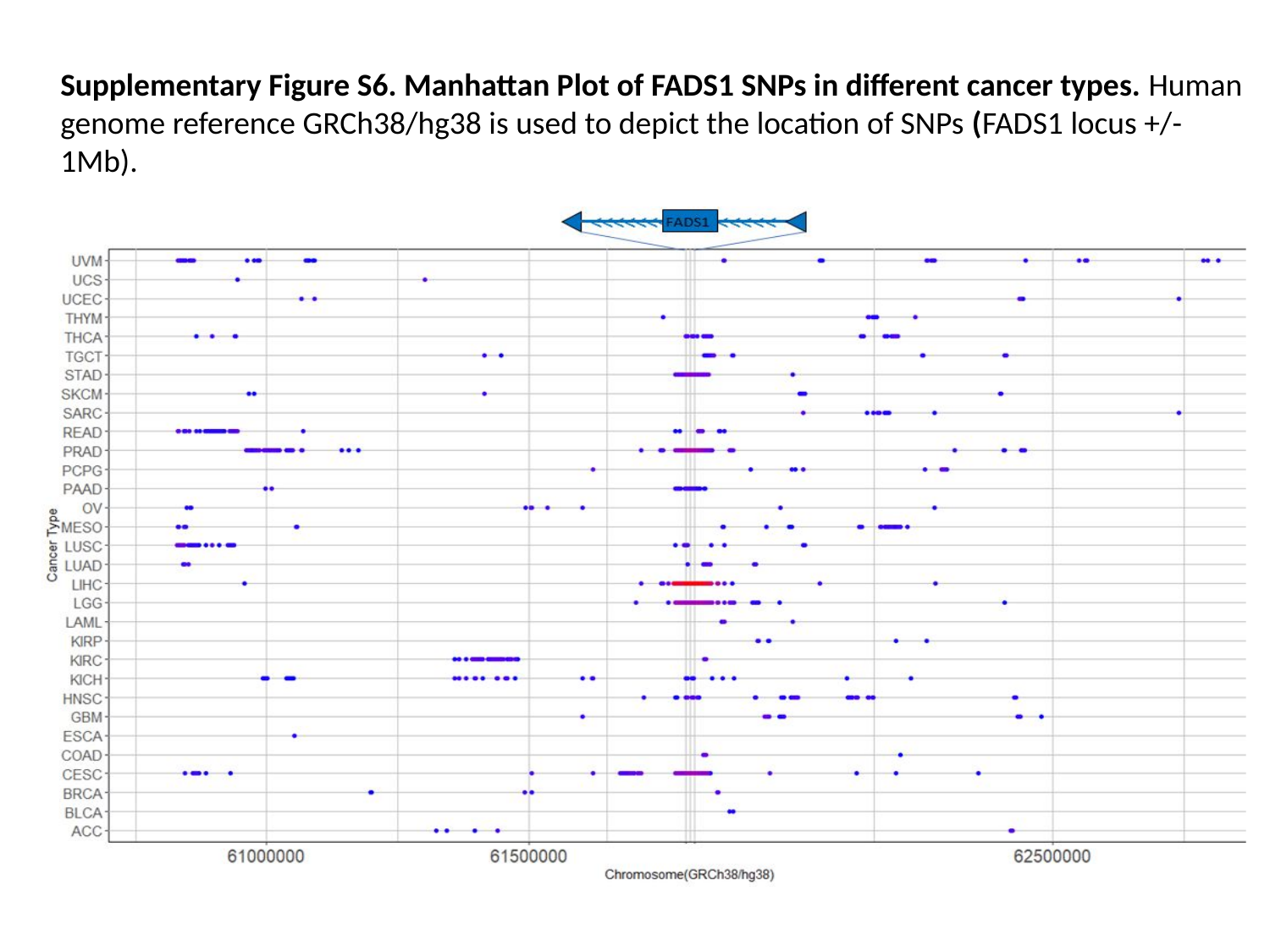

Supplementary Figure S6. Manhattan Plot of FADS1 SNPs in different cancer types. Human genome reference GRCh38/hg38 is used to depict the location of SNPs (FADS1 locus +/- 1Mb).

## Slide 27
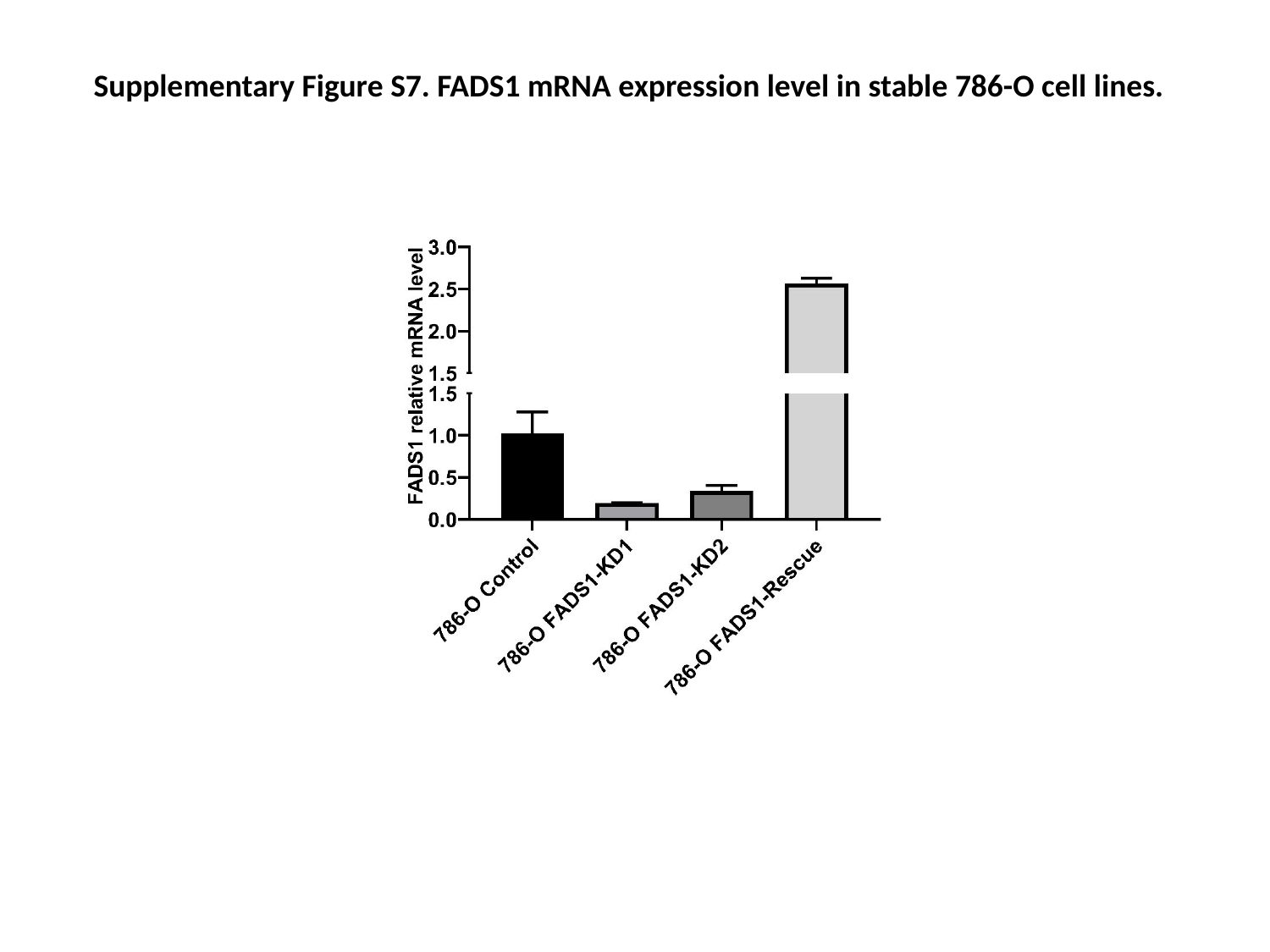

Supplementary Figure S7. FADS1 mRNA expression level in stable 786-O cell lines.

## Slide 28
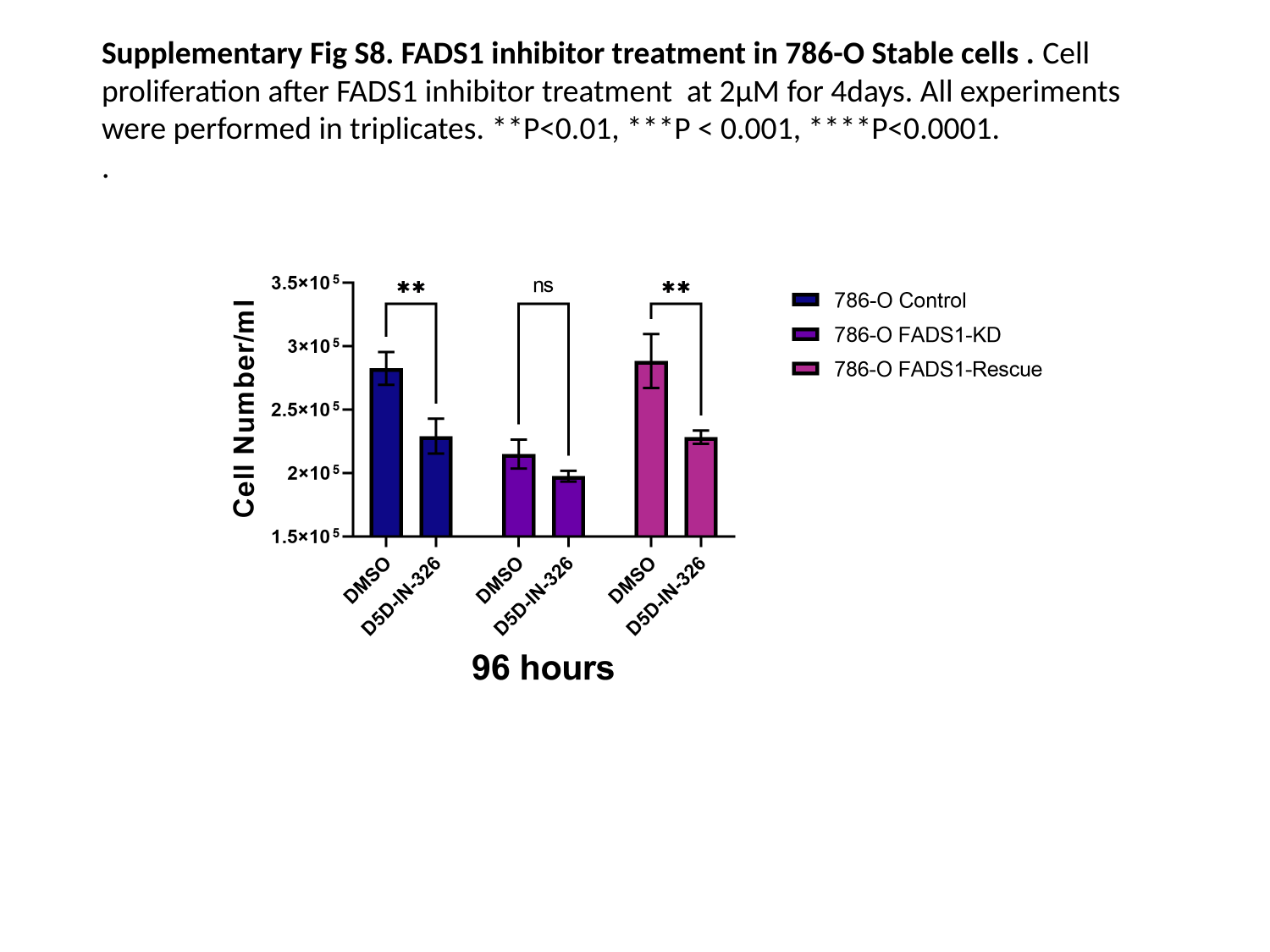

Supplementary Fig S8. FADS1 inhibitor treatment in 786-O Stable cells . Cell proliferation after FADS1 inhibitor treatment at 2µM for 4days. All experiments were performed in triplicates. **P<0.01, ***P < 0.001, ****P<0.0001.
.

## Slide 29
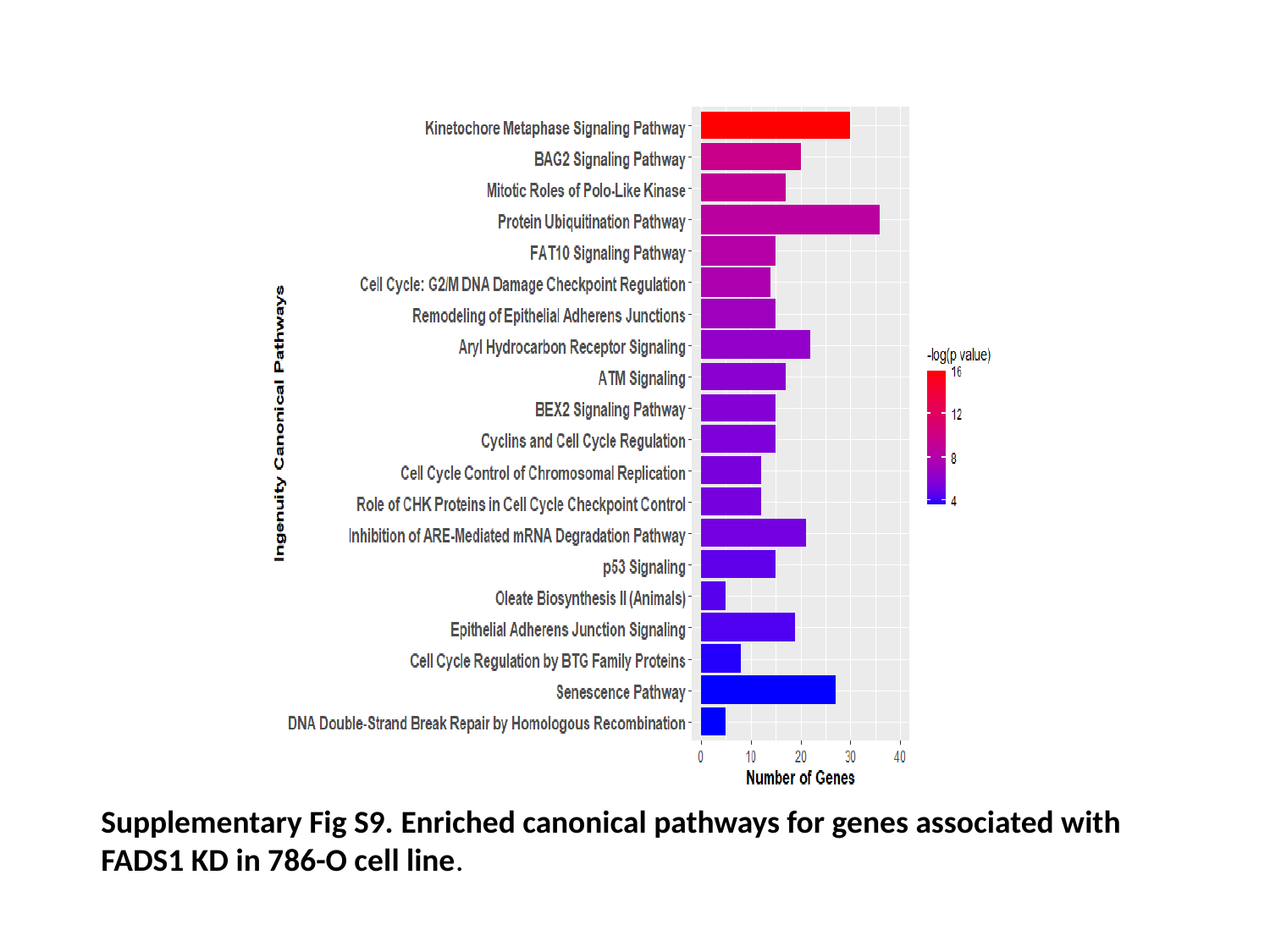

Supplementary Fig S9. Enriched canonical pathways for genes associated with FADS1 KD in 786-O cell line.
